# Decitabine- and 5-azacytidine resistance emerges from adaptive responses of the pyrimidine metabolism network

**DOI:** 10.1038/s41375-020-1003-x

**Published:** 2020-08-07

**Authors:** Xiaorong Gu, Rita Tohme, Benjamin Tomlinson, Nneha Sakre, Metis Hasipek, Lisa Durkin, Caroline Schuerger, Dale Grabowski, Asmaa M. Zidan, Tomas Radivoyevitch, Changjin Hong, Hetty Carraway, Betty Hamilton, Ronald Sobecks, Bhumika Patel, Babal K. Jha, Eric D. Hsi, Jaroslaw Maciejewski, Yogen Saunthararajah

**Affiliations:** 1 Department of Translational Hematology & Oncology Research, Taussig Cancer Institute, Cleveland Clinic, Cleveland, OH USA; 2 Department of Hematology and Oncology, University Hospitals, Cleveland, OH USA; 3 Department of Clinical Pathology, Tomsich Pathology Institute, Cleveland Clinic, Cleveland, OH USA; 4 Department of Quantitative Health Sciences, Cleveland Clinic, Cleveland, OH USA; 5 Department of Hematology and Oncology, Taussig Cancer Institute, Cleveland Clinic, Cleveland, OH USA

**Keywords:** Cancer therapy, Translational research

## Abstract

Mechanisms-of-resistance to decitabine and 5-azacytidine, mainstay treatments for myeloid malignancies, require investigation and countermeasures. Both are nucleoside analog pro-drugs processed by pyrimidine metabolism into a deoxynucleotide analog that depletes the key epigenetic regulator DNA methyltranseferase 1 (DNMT1). Here, upon serial analyses of DNMT1 levels in patients’ bone marrows on-therapy, we found DNMT1 was not depleted at relapse. Showing why, bone marrows at relapse exhibited shifts in expression of key pyrimidine metabolism enzymes in directions adverse to pro-drug activation. Further investigation revealed the origin of these shifts. Pyrimidine metabolism is a network that senses and regulates deoxynucleotide amounts. Deoxynucleotide amounts were disturbed by single exposures to decitabine or 5-azacytidine, via off-target depletion of thymidylate synthase and ribonucleotide reductase respectively. Compensating pyrimidine metabolism shifts peaked 72–96 h later. Continuous pro-drug exposures stabilized these adaptive metabolic responses to thereby prevent DNMT1-depletion and permit exponential leukemia out-growth as soon as day 40. The consistency of the acute metabolic responses enabled exploitation: simple treatment modifications in xenotransplant models of chemorefractory leukemia extended noncytotoxic DNMT1-depletion and leukemia control by several months. In sum, resistance to decitabine and 5-azacytidine originates from adaptive responses of the pyrimidine metabolism network; these responses can be anticipated and thus exploited.

## Graphical abstract

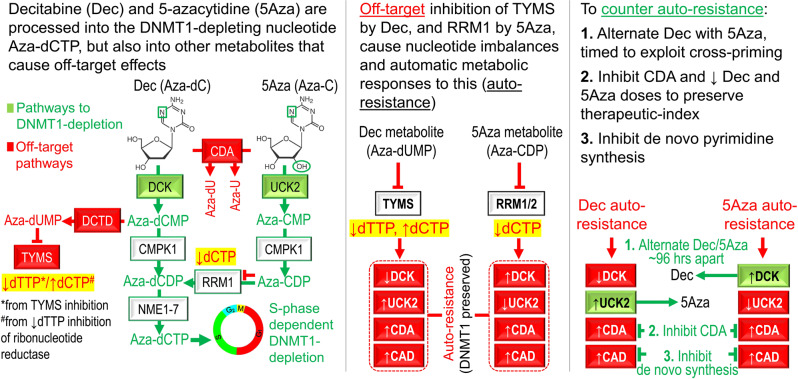

## Introduction

The deoxycytidine analog pro-drug decitabine and the cytidine analog pro-drug 5-azacytidine can increase life-spans of patients with myeloid malignancies, shown by randomized trials in patients with myelodysplastic syndromes (MDS) and acute myeloid leukemia (AML)(reviewed in [1]). Both pro-drugs are processed by pyrimidine metabolism into a deoxycytidine triphosphate (dCTP) analog, Aza-dCTP, that depletes the key epigenetic regulator DNA methyltransferase 1 (DNMT1) from dividing cells [2]. DNMT1-depletion terminates malignant self-replication but maintains normal hematopoietic stem cell self-replication [3–12]—a vital therapeutic index when treating myeloid malignancies, since normal hematopoiesis is needed to reverse low blood counts, the cause of morbidity and death. Moreover, the cancer cell cycling exits triggered by DNMT1-depletion do not require the p53 apoptosis axis—this is an important contrast with conventional anti-metabolite chemotherapy—even patients with high-risk, *TP53*-mutated, chemorefractory disease can benefit from decitabine or 5-azacytidine therapy (reviewed in [1, 13]). Even so, only ~40% of treated patients benefit overall, and even in responders, relapse is routine. There is therefore a need to understand the mechanisms by which malignant cells resist decitabine or 5-azacytidine, and to use such knowledge to improve response rates and durations. An important piece of this puzzle could be how these pro-drugs are processed into DNMT1-targeting Aza-dCTP.

Decitabine and 5-azacytidine have an identical modification in the pyrimidine ring—replacement of carbon at position 5 with nitrogen—but the sugar moiety is deoxyribose in decitabine and ribose in 5-azacytidine. This channels their metabolism differently, with the following enzymes having central roles: deoxycytidine kinase (DCK), uridine cytidine kinase 2 (UCK2), cytidine deaminase (CDA), and carbamoyl-phosphate synthetase (CAD). DCK phosphorylates decitabine, the rate-limiting step in its processing toward Aza-dCTP [14]. *DCK*-null AML cells thus resisted decitabine, even at a concentration of 360 µM [15], and sensitivity was restored by transfection with an expression vector for DCK [14, 16]. On the other hand, 5-azacytidine is phosphorylated by UCK2 [17, 18]. Thus, AML cell lines resistant to >50 µM of 5-azacytidine contained inactivating mutations in *UCK2* [19], and sensitivity was restored by transfection with an expression vector for UCK2 [19]. Despite such in vitro data, contributions of altered DCK and/or UCK2 to clinical relapse have been minimally investigated: one study of 14 decitabine-treated patients measured DCK expression in peripheral blood or bone marrow at relapse versus diagnosis, with inconclusive results [20]; another study of eight decitabine-treated patients did find that DCK expression was significantly decreased at relapse [21].

The pyrimidine metabolism enzymes CDA and CAD have also been shown to contribute to resistance to decitabine and 5-azacytidine in vitro, via catabolism and competition respectively: CDA rapidly catabolizes both pro-drugs into uridine counterparts that do not deplete DNMT1 [22] and that instead may cause off-target antimetabolite effects, e.g., by misincorporating into DNA [23]. Expression vectors for CDA therefore conferred decitabine-resistance to malignant cells [24, 25], as did CDA-rich tissue micro-environments (e.g., liver) [26]. Naturally high CDA expression in liver/gastro-intestinal tract is also why decitabine and 5-azacytidine have brief plasma half-lives of <15 min with parenteral administration and trivial oral bioavailability [27]. In clinical analyses, poorer outcomes with decitabine or 5-azacytidine treatment of male versus female MDS patients was linked to sex-differences in CDA expression [28–30]. CAD is the first enzyme in the de novo pathway that synthesizes pyrimidine nucleotides from glutamine and aspartate: de novo synthesized dCTP can compete with Aza-dCTP for incorporation into DNA, and accordingly, CAD upregulation has also been implicated in resistance to 5-azacytidine in vitro [17, 31, 32].

Altogether therefore, DCK, UCK2, CDA, and CAD expression changes are known to mediate resistance to decitabine or 5-azacytidine in vitro, but there is little information and no countermeasures for their individual or collective contributions to clinical resistance. Here, upon a first serial analyses of DNMT1 levels in patients’ bone marrows on-therapy, we found that this target was not being engaged at clinical relapse. Indeed, bone marrows at relapse exhibited shifts in DCK, UCK2, CDA, and CAD expression in directions adverse to pro-drug conversion into DNMT1-depleting Aza-dCTP. Pyrimidine metabolism is a network that senses and regulates deoxynucleotide amounts [33]: we found that decitabine and 5-azacytidine cause distinct deoxynucleotide imbalances via off-target depletion of thymidylate synthase and ribonucleotide reductase respectively, to in turn trigger specific, compensatory changes in expression of key pyrimidine metabolism enzymes. The consistency and predictability of metabolic reconfiguration enabled anticipation, out-maneuvering and even exploitation: simple, practical treatment modifications preserved the favorable therapeutic index of noncytotoxic DNMT1-depletion and markedly improved efficacy in preclinical in vivo models of aggressive chemo-refractory AML.

## Methods

### Study approvals

Bone marrow samples, and primary AML cells for inoculation into NSG mice, were collected with written informed consent on Cleveland Clinic Institutional Review Board approved protocols (Cleveland, OH) from all patients. Murine experiments were in accordance with a protocol approved by the Cleveland Clinic Institutional Animal Care and Use Committee (Cleveland, OH).

### Statistics

Assuming a rate of lethal AML in vehicle-treated mice to be 100% versus 30% with drug-treatment, 6 weeks after AML cell inoculation, comparing these proportions with an alpha of 0.05 and single-sided power of 0.8, the required sample size in each group with equal allocation was seven (Fishers Exact method (https://stattools.crab.org/). If early data indicated larger treatment effect sizes, subsequent sample sizes were reduced to 5 mice per treatment group, in accordance with refine, reduce, replace principles.

Tumor burdens were compared using nonparametric tests, and survival curves by the Log-rank test. Wilcoxon rank sum, Mann Whitney, and *t* tests were two-sided unless confirming prior literature observations (dCTP level analyses) and performed at the 0.05 significance level or lower (Bonferroni corrections were applied for instances of multiple parallel testing). Standard deviations and inter-quartile ranges for each set of measurements were calculated and represented as *y*-axis error bars on each graph. Data-points/distributions are from biological replicates for in vitro experiments, and from individual patients/animals in vivo.

Graph Prism (GraphPad, San Diego, CA) or SAS statistical software (SAS Institute Inc., Cary, NC) was used to perform statistical analysis including correlation analyses. Detailed method in “Supplementary information”.

## Results

### DNMT1 is not depleted with clinical or in vitro resistance

**Fig. 1:**
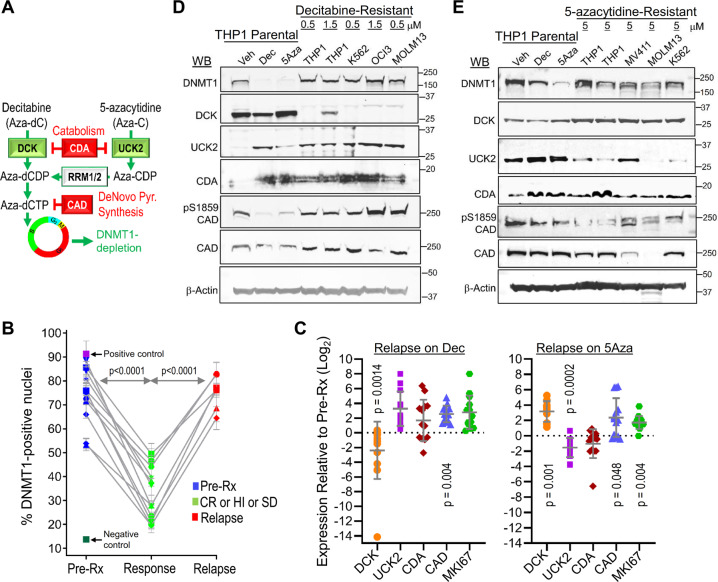
DNMT1 is not depleted at clinical or in vitro resistance. **a** Schema shows key pyrimidine metabolism enzymes that favor (green) or impede (red) decitabine (Dec) or 5-azacytidine (5Aza) conversion into DNMT1-depleting Aza-dCTP. dCDP = deoxycytidine diphosphate; CDP = cytidine diphosphate; dCTP—deoxycytidine triphosphate. **b** Dec or 5Aza decreased bone marrow DNMT1 at clinical response (green) versus pretreatment (dark blue) but not at relapse (red). Serial bone marrow biopsies from the same patent were cut onto the same slide, stained for DNMT1, and the number of DNMT1-positive nuclei was quantified objectively using ImageIQ software (*n* = 13 patients; positive/negative controls were wild-type and *DNMT1*-knockout HCT116 tissue blocks respectively). Pre-R_x_ = pretreatment; HI = hematologic improvement; CR = complete remission; SD = stable disease. Mean ± SD of ≥3 image segments (cellular regions) per sample; *p* value paired *t*-test, two-sided. **c** Pyrimidine metabolism enzyme expression at relapse on Dec or 5Aza. Bone marrow cells aspirated pretreatment and at relapse/progression on Dec (13 patients, median duration of therapy 175 days, range 97–922) or 5Aza (14 patients, median duration of therapy 433 days, range 61–1155) were analyzed by QRT-PCR. Mean ± SD, paired *t*-test, two-sided. **d**, **e** DNMT1 and pyrimidine metabolism enzyme protein expression in Dec- or 5Aza-resistant AML cells. We selected for AML cells THP1, K562, MOLM13, and OCI-AML3 or MV411 growing exponentially through Dec or 5Aza at the indicated concentrations. Parental THP1 AML cells treated with vehicle, Dec 0.25 µM or 5Aza 2.5 µM were included for comparison purposes. Primary antibodies for P-S1859 and total CAD were both rabbit and thus probed on separate gels/blots. CDA analysis was in nuclear fractions. Equal loading was confirmed for all Western blots.

Serial bone marrow biopsies from the same patient, before and during therapy with decitabine or 5-azacytidine, were cut onto the same glass slide and stained simultaneously to facilitate time-course comparison of DNMT1 protein quantified by immunohistochemistry and ImageIQ imaging/software (39 serial bone marrow samples from 13 patients, median treatment duration 372 days, range 170–1391) (Fig. 1a, b). At time-of-response, DNMT1 protein was significantly decreased by ~50% compared to pretreatment (Fig. 1b). At the time-of-relapse on-therapy, however, DNMT1 protein rebounded to levels comparable to pretreatment (Fig. 1b).

Since DNMT1-depletion by decitabine and 5-azacytidine in vitro is well-documented to be impacted by expression levels of the pyrimidine metabolism enzymes DCK, UCK2, CDA, and CAD (Fig. 1a), we measured expression of these enzymes, by quantitative polymerase chain reaction (QRT-PCR), in MDS patients’ bone marrows pretreatment and at relapse on-therapy with decitabine (*n* = 13, median treatment-duration 175 days, range 97–922) or 5-azacytidine (*n* = 14, median treatment-duration 433 days, range 61–1155) (Fig. 1c). DCK expression was approximately halved at relapse on decitabine versus pretreatment but increased by approximately eightfold at relapse on 5-azacytidine (Fig. 1c). UCK2 expression was a mirror-image: approximately eightfold increased at relapse on decitabine and approximately halved at relapse on 5-azacytidine (Fig. 1c). CDA expression increased approx threefold at relapse on decitabine and approximately halved at relapse on 5-azacytidine (Fig. 1c). CAD increased up to approximately eight fold at relapse on either pro-drug, but not in all patients (Fig. 1c). The proliferation marker MKI67 increased at relapse in almost all the patients, consistent with active progression of disease (Fig. 1c).

We then evaluated resistance to decitabine or 5-azacytidine in vitro: AML cells (THP1, K562, MOLM13 and OCI-AML3 or MV411) were cultured in the presence of decitabine 0.2–1.5 μM or 5-azacytidine 2–5 μM (clinically relevant concentrations). After initial cytoreductions, AML cells proliferating exponentially through decitabine or 5-azacytidine emerged as early as day 40 (Supplementary Fig. S1). DNMT1 was not depleted from these decitabine- or 5-azacytidine-resistant AML cells (Fig. 1d, e). DCK protein was suppressed with decitabine-resistance (Fig. 1d) but upregulated in 5-azacytidine resistant cells (Fig. 1e). UCK2 protein was upregulated in decitabine-resistant but suppressed in 5-azacytidine-resistant cells (Fig. 1d, e). CDA protein was upregulated in decitabine-resistant and to a lesser extent in 5-azacytidine-resistant cells (Fig. 1d, e). CAD was upregulated by total protein and serine 1856 phosphorylation (post-translational modification linked with its functional activation) in decitabine-resistant but not in 5-azacytidine-resistant cells (Fig. 1d, e). 5-azacytidine depleted DNMT1 and cytoreduced decitabine-resistant AML cells (Supplementary Fig. S2). Thus, clinical and in vitro resistance to decitabine or 5-azacytidine were characterized by preserved DNMT1, mirror-image shifts in DCK and UCK2, and upregulation of CDA and CAD most consistently with resistance to decitabine.

### Decitabine and 5-azacytidine cause acute deoxynucleotide imbalances and metabolic compensations

**Fig. 2:**
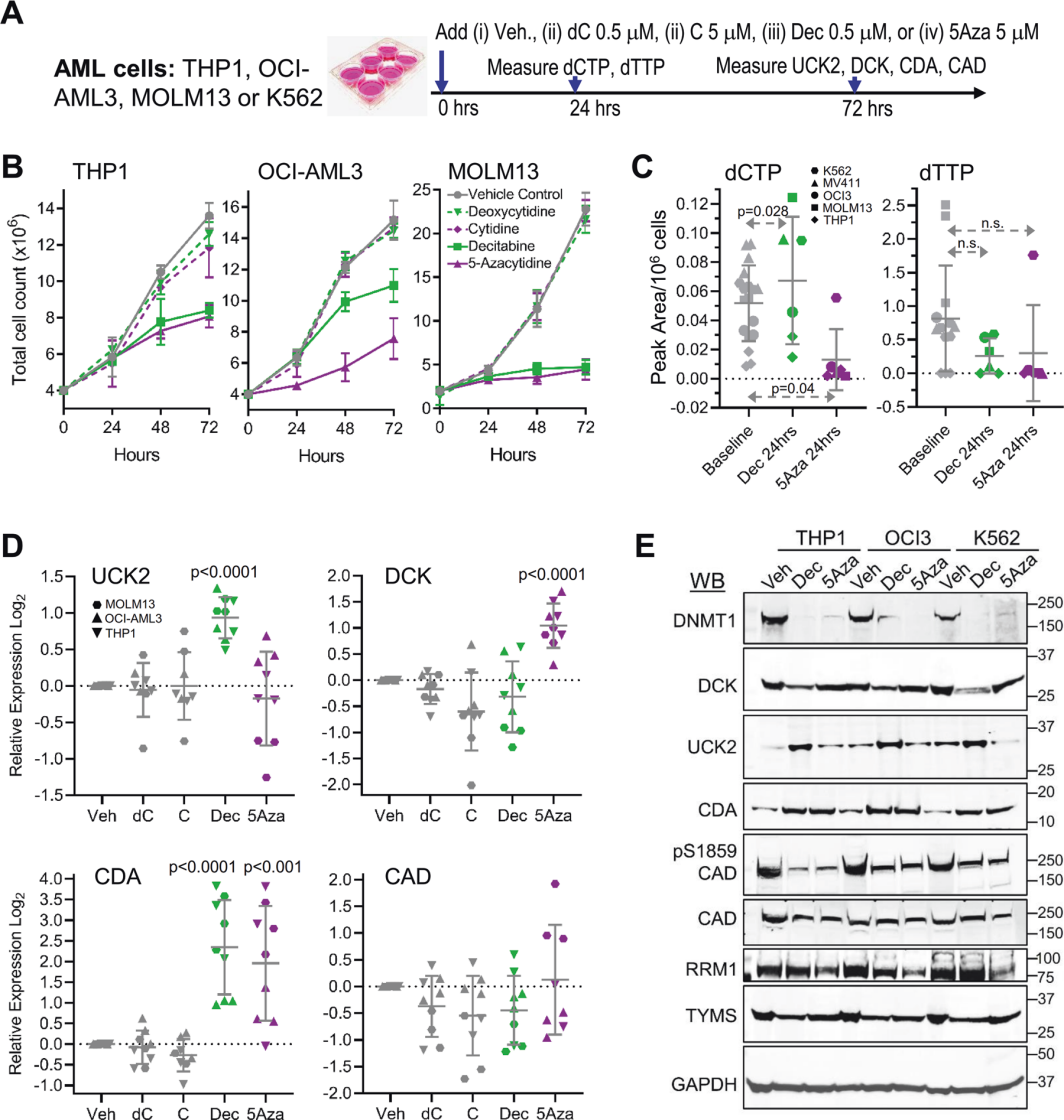
Dec and 5Aza cause nucleotide imbalances and automatic metabolic compensations for this. **a** Experiment schema. Vehicle, natural deoxycytidine (dC) 0.5 μM, natural cytidine (C) 5 μM, Dec 0.5 μM, or 5Aza 5 μM were added once to AML cells at 0 h. **b** Cell counts. By automated counter. Means ± SD for three independent biological replicates for each cell line. **c** Dec and 5Aza have opposite effects on dCTP levels. Measured by LCMS/MS 24 h after addition of Dec or 5Aza. Analyses of two or more independent nucleotide extractions from three different AML cells lines. Means ± SD; *p* values paired *t*-test, one-sided. **d** Gene expression 72 h after Dec or 5Aza. Gene expression by QRT-PCR, relative to average expression in vehicle-treated controls. Means ± SD for three independent biological replicates in each of three AML cell lines; *p* values unpaired *t*-test versus vehicle, two-sided. **e** Western blot 72 h after Dec or 5Aza. AML cells THP1, OCI-AML3, and K562. Western blots were reproduced in three independent biological replicates.

We examined whether decitabine and 5-azacytidine disequilibrate deoxynucleotides to thereby trigger compensatory metabolic responses. AML cells (MOLM13, OCI-AML3, THP1) were treated with a single dose of vehicle, natural deoxycytidine 0.5 µM, decitabine 0.5 µM, natural cytidine 5 µM, or 5-azacytidine 5 µM in vitro, and effects on dCTP and deoxythymidine triphosphate (dTTP) levels, and pyrimidine metabolism gene expression, were measured 24–72 h later (Fig. 2a).

Vehicle, deoxycytidine and cytidine did not impact proliferation of the AML cells (Fig. 2b). A single treatment with either decitabine or 5-azacytidine, on the other hand, significantly decreased AML cell proliferation (Fig. 2b). We then measured impact on dCTP and dTTP amounts at 24 h: dCTP was significantly increased by decitabine but significantly decreased by 5-azacytidine (Fig. 2c). dTTP was decreased by both pro-drugs, but not to statistical significance (Fig. 2c). We then measured expression of key pyrimidine metabolism enzymes; serial Western blot measurements indicated peak protein expression changes occurred 48–96 h after addition of pro-drug (Supplementary Fig. S3A,B). We thus focused repeat measurements at 72 h, using both QRT-PCR and Western blots. DCK mRNA and protein levels were significantly increased by the single addition of 5-azacytidine but not decitabine (Figs. 2d, e, Supplementary Fig. S3). Conversely, UCK2 mRNA and protein levels were significantly increased by decitabine but not 5-azacytidine (Figs. 2d, e, Supplementary Fig. S3). CDA mRNA and protein levels were significantly increased by both pro-drugs (Figs. 2d, e, Supplementary Fig. S3). Neither pro-drug changed total CAD or cytidine triphosphate synthetase 1 (CTPS1) levels (CTPS1 executes a late step in de novo pyrimidine synthesis) (Figs. 2d, e, Supplementary Fig. S3). Both pro-drugs did, however, decrease phosphorylation of CAD at serine 1856 (Fig. 2e). Both decitabine and 5-azacytidine depleted DNMT1 as expected (Figs. 2e, Supplementary Fig. S3).

We extended protein level analyses to additional pyrimidine metabolism enzymes playing nodal roles in nucleotide balance: thymidylate synthase (TYMS) that is the major mediator of dTTP production [34–36], and sub-units RRM1 and RRM2A of the ribonucleotide reductase complex that converts RNA molecules, such as 5-azacytidine (after diphosphorylation), into DNA molecules such as decitabine. TYMS was depleted by both pro-drugs, but to a noticeably greater extent by decitabine than 5-azacytidine (Fig. 2e, Supplementary Fig. S3)—the natural substrate of TYMS is deoxyuridine monophosphate (dUMP), and decitabine and 5-azacytidine are metabolized into a dUMP analog Aza-dUMP by 2 versus 6 catalytic steps respectively (Supplementary Fig. S3C). 5-azacytidine, but not decitabine, depleted RRM1 (Fig. 2e), with less impact on the ribonucleotide reductase sub-unit RRM2A (Supplementary Fig. S3).

### DCK and UCK2 are important for maintaining dCTP and dTTP, respectively

**Fig. 3:**
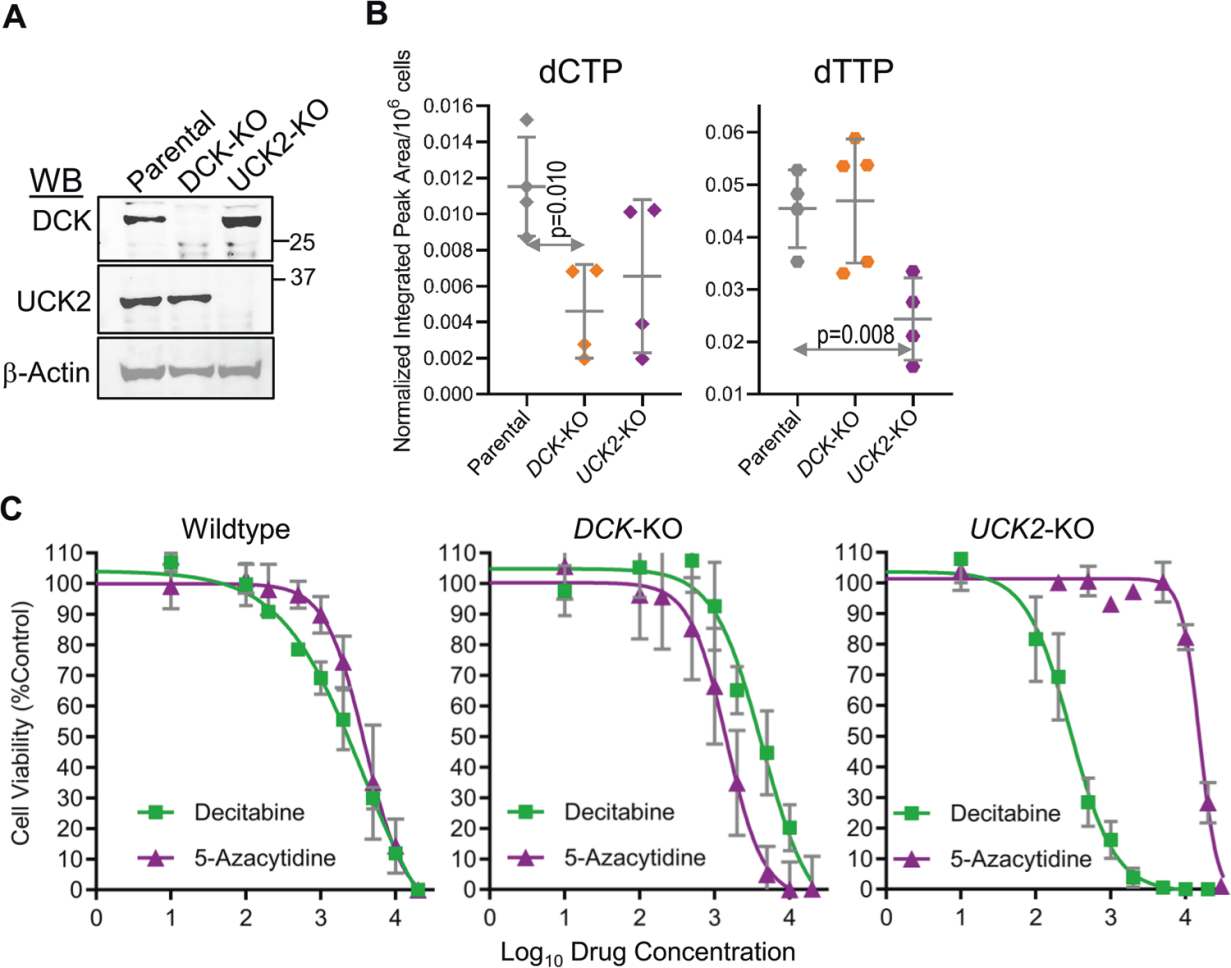
DCK is important for maintaining dCTP and UCK2 for maintaining dTTP levels. **a** DCK and UCK2 knockout (KO) were confirmed by Western blot. HAP1 leukemia cells, KO by CRISPR-Cas9. **b** *DCK*-KO lowers dCTP and *UCK2*-KO lowers dTTP. Analysis of independent nucleotide extractions. Means ± SD; *p* values unpaired *t*-test, two-sided. **c** Sensitivity of Wildtype, *DCK*-KO and *UCK2*-KO HAP1 leukemia cells to Dec versus 5Aza. Means ± SD of three independent biological replicates.

To better understand contributions of DCK and UCK2 to dCTP and dTTP maintenance, we knocked DCK and UCK2 out of leukemia cells (HAP1) using CRISPR-Cas9 then measured levels of the deoxynucleotides. *DCK*-knockout, but not *UCK2*-knockout, significantly decreased dCTP (Fig. 3a, b). Thus, DCK appears important to dCTP maintenance, consistent with DCK upregulation as an appropriate compensatory response to dCTP suppression by 5-azacytidine (Fig. 2). *UCK2*-knockout, but not *DCK*-knockout, significantly decreased dTTP (Fig. 3a, b). Thus, UCK2 appears important to dTTP maintenance, consistent with UCK2 upregulation as a response to dTTP suppression by decitabine (Fig. 2).

We also examined sensitivity of the *DCK*- and *UCK2*-knockout cells to decitabine and 5-azacytidine. *DCK*-knockout cells were relatively resistant to decitabine (concentrations for 50% growth inhibition [GI50] 12 versus 3 μM for parental cells), but more sensitive to 5-azacytidine (GI50 2 versus 4 μM for parental cells) (Fig. 3c). *UCK2*-knockout cells were relatively resistant to 5-azacytidine (GI50 15 μM versus 4 μM for parental cells), but more sensitive to decitabine (GI50 0.1 μM versus 3 μM for parental cells) (Fig. 3c).

### Resistance countermeasures evaluated in vivo

We then evaluated potential solutions for resistance (summarized in Table 1) in a patient-derived xenotransplant model (PDX) of AML that was chemorefractory to both decitabine and cytarabine. Initial experiments used AML burden as the primary end-point, quantified by flow cytometry of bone marrow, complemented by spleen weights, measured at a fixed time-point after initiation of therapy. Important/promising results found this way were evaluated in a second phase of experiments that used time-to-distress/survival as the primary end-point: treatment was continued until a mouse showed signs of distress as defined in our Animal Protocol at which point the individual mouse was euthanized.

**Table 1:** Solutions for resistance to decitabine and 5-azacytidine evaluated in vivo.

	Potential solution	Rationale	Results
1.	↑Dose	– Overwhelm resistance mediated by DCK, UCK2, CDA and CAD expression changes	Cytotoxic to normal sensitive tissues (bone marrow) but failed to deplete DNMT1 in malignant tissues with ↓DCK, ↑CDA and ↑CAD [3, 26] (poor therapeutic index)
2.	Frequent, distributed schedules of administration	– ↑ Overlap between drug exposure windows and malignant S-phase entries –- Avoid troughs in DCK/UCK2 expression induced by the pro-drugs	(a) Frequent, distributed administration of Dec 2–3X/week was superior to pulse-cycled administration for 5 consecutive days/month (Supplementary Fig. S6) (b) Dec scheduled to avoid DCK troughs (Day 1,2 each week) was superior to scheduling that coincided with DCK troughs (Day 1,4 each week) (Supplementary Figs. S4, S5)
3.	Add tetrahydrouridine (THU)(CDA-inhibitor)	– Overcome CDA-imposed limits on Dec/5Aza plasma t_½_ and tissue distribution – Overcome CDA-imposed limits on Dec/5Aza intra-cellular t_½_ – Counter auto-upregulation of CDA by Dec	(a) THU increased Dec or 5Aza systemic bioavailability approximately tenfold [40, 41] and increased intra-cellular t_½_ [57–59] (b) THU + Dec or THU + 5Aza (Dec/5Aza doses reduced by 50%) was superior to Dec or 5Aza alone (Figs. 4–6)
4.	Add hydroxyurea or thymidine (ribonucleotide reductase inhibitors)	– Counter competition from dCTP produced via de novo pyrimidine synthesis	Hydroxyurea or thymidine did not add benefit to Dec or THU/Dec (Fig. 4, Supplementary Fig. S6).
5.	Alternate Dec with 5Aza, timed to exploit reactive peaks in DCK and UCK2 expression	– Dec primes for 5Aza uptake and vice versa, effects that peak at 72–96 h	THU + Dec/THU + 5Aza alternated week to week (Fig. 6) was superior to THU + Dec or THU + 5Aza alone (Figs. 5, Supplementary Fig. S7), or THU + Dec/THU + 5Aza alternated month to month (Fig. 5) or given simultaneously (Supplementary Fig. S7)

(a) *Schedule decitabine administration to avoid DCK troughs*: Immune-deficient mice were tail-vein innoculated with 1 million human AML cells each. On Day 9 after innoculation, mice were randomized to treatment with (i) vehicle; (ii) decitabine timed to avoid DCK troughs (Day 1 and Day 2 each week—Day 1,2); or (iii) decitabine timed to coincide with DCK troughs (Day 1 and Day 4 each week—Day 1,4) (Supplementary Fig. S4A). Vehicle-treated mice showed distress on day 45 of treatment, at which point all mice were sacrificed for analyses. The bone marrows of vehicle and Day 1,4 but not Day 1,2 treated mice, were replaced by AML cells observed by microscopy (Supplementary Fig. S4B) and by flow cytometry: human CD45+ (hCD45+) AML cells were ~92% with PBS, ~63% with Day 1,4 and ~26% with Day 1,2 treatment (Supplementary Fig. S4C). Spleens were enlarged with effaced histology by AML with vehicle or Day 1,4 but had mostly preserved histology with Day 1,2 treatment (Supplementary Fig. S4D, E). Spleen weights as another measure of AML burden were also lowest with Day 1,2 treatment (Supplementary Fig. S4F).

These two schedules of decitabine administration were compared again but with waiting for signs of distress in individual mice rather than collective sacrifice at day 45 (Supplementary Fig. S5A). Median survival (time-to-distress) was significantly better with Day 1,2 (75 days) versus Day 1,4 (60 days) or vehicle treatment (40 days) (Supplementary Fig. S5B), and bone marrow and spleen AML burden was again lowest with Day 1,2 versus Day 1,4 or vehicle treatment, despite the later day of euthanasia for the Day 1,2 mice (Supplementary Fig. S5C–E).

Thus, scheduling decitabine administration to avoid DCK troughs (Day 1,2) was superior to scheduling that coincided with these troughs (Day 1,4).

**Fig. 4:**
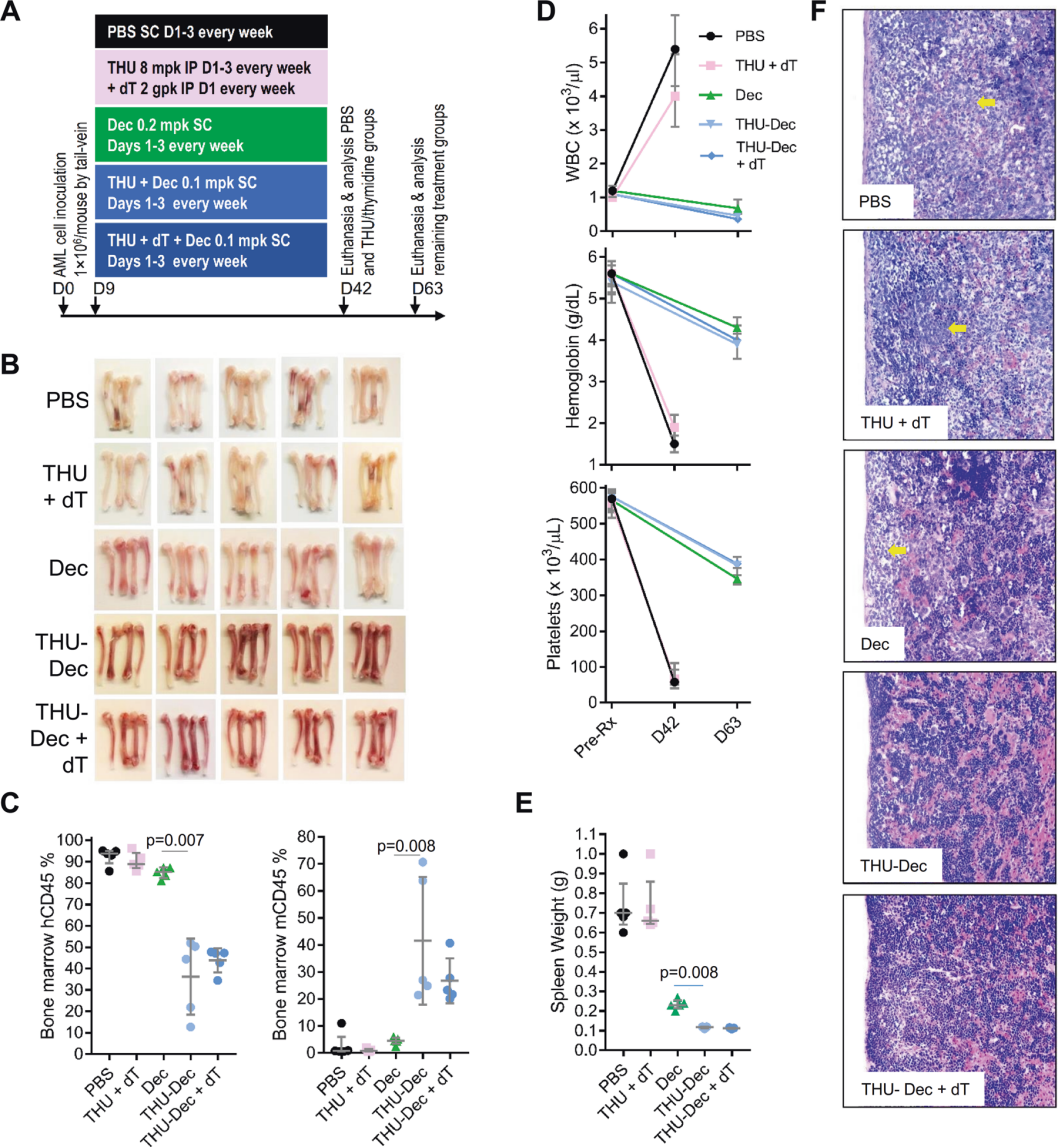
Impact on efficacy of adding inhibitors of CDA and/or de novo pyrimidine synthesis. NSG mice were tail-vein inoculated with patient-derived AML cells (1 × 10^6^ cells/mouse) and randomized to (i) PBS vehicle; (ii) CDA-inhibitor (intra-peritoneal [IP] THU)+ de novo pyrimidine synthesis inhibitor (IP thymidine [dT]); (iii) Dec; (iv) THU + Dec; (v) THU + Dec + dT (*n* = 5/group). PBS and THU + dT mice were euthanized for distress on D42, and other mice were sacrificed for analysis on D63. **a** Experiment schema. **b** Femoral bones. White = leukemia replacement, reddish = functional hematopoiesis. **c** Bone marrow human (hCD45) and murine (mCd45) myelopoiesis content. Flow-cytometry. Median ± IQR. *p* value Mann–Whitney test two-sided. **d** Blood counts pretreatment and at euthanasia/sacrifice. Measured by Hemavet. Median ± IQR. **e** Spleen AML burden (spleen weights) at euthanasia/sacrifice. Median ± IQR. *p* value Mann–Whitney test two-sided. **f** Spleen histology. Hematoxylin-Eosin stain of paraffin-embedded sections. Magnification ×400. Leica DMR microscope.

**Fig. 5:**
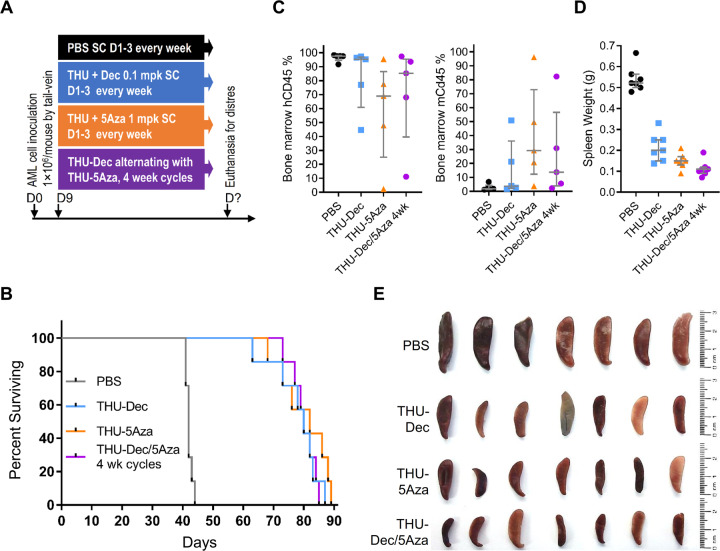
Comparison of THU + Dec alone versus THU + 5Aza alone versus THU + Dec alternating with THU + 5Aza in 4 week cycles. NSG mice were tail-vein inoculated with patient-derived AML cells (1 × 10^6^ cells/mouse) and on Day 9 after inoculation randomized to the treatments as shown (*n* = 7/group). Mice were euthanized if there were signs of distress. **a** Experiment schema; **b** Time-to-distress and euthanasia. **c** Bone marrow human (hCD45) and murine (mCd45) myelopoiesis content. Femoral bones flushed after euthanasia. Measured by flow-cytometry. Median ± IQR. **d** Spleen weights at time-of-distress/euthanasia. Median ± IQR. **e** Spleens at the time-of-distress/euthansia.

**Fig. 6:**
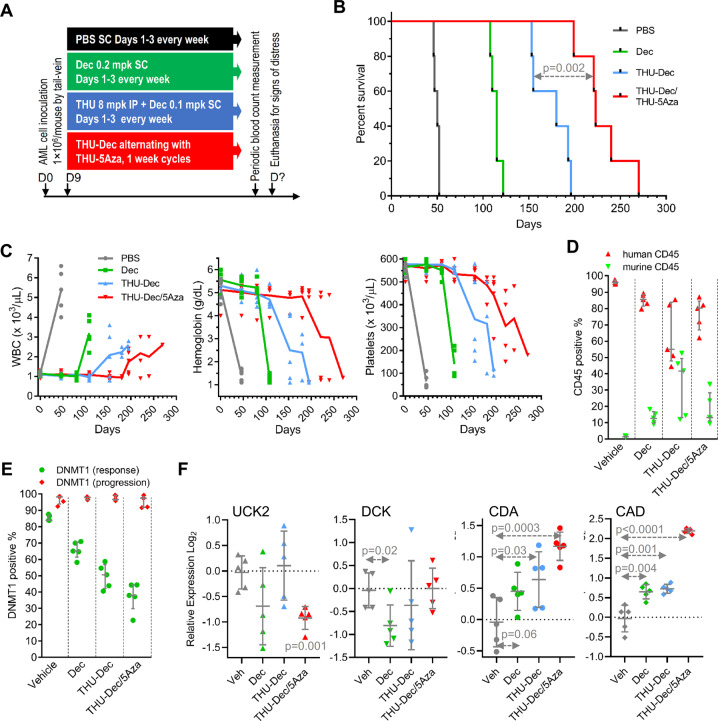
Alternating THU + Dec with THU + 5Aza week to week. NSG mice were tail-vein inoculated with patient-derived AML cells (1 × 10^6^ cells/mouse) and randomized to the treatments shown (*n* = 5/group). Blood counts were obtained periodically by tail-vein phlebotomy. Mice were euthanized for signs of distress. **a** Experiment schema; **b** Time-to-distress. Log-rank test. **c** Serial blood counts. Measured by Hemavet. Median ± IQR. **d** Bone marrow replacement by AML. Bone marrow human and murine CD45 + cells measured by flow-cytometry after euthanasia (time-points **b**). Median ± IQR. *p* value Mann–Whitney test two-sided. **e** DNMT1 was not depleted from AML cells at progression (timepoints **b**) but was depleted at time-of-response (bone marrow harvested at Day 63 in a separate experiment). Flow cytometry. **f** Pyrimidine metabolism gene expression in bone marrow AML cells. QRT-PCR using human gene specific primers, bone marrow harvested after euthanasia. *p* values versus vehicle, unpaired *t*-test, two-sided.

(b) *Combine with CDA and/or ribonucleotide reductase inhibitors*: CDA can be inhibited by tetrahydrouridine (THU), while de novo pyrimidine synthesis can be inhibited at ribonucleotide reductase using deoxythymidine (dT) [37]. NSG mice tail-vein innoculated with 1 million AML cells each were randomized to (i) vehicle; (ii) THU + dT, (iii) decitabine; (iv) THU + decitabine; or (v) THU + dT + decitabine (Fig. 4a). PBS and THU + dT-treated mice developed signs of distress, and were euthanized, on day 42. To increase chances of seeing differences in AML burden between other treatments, other mice were sacrificed 3 weeks later on day 63 (Fig. 4a). Visual inspection and flow cytometry demonstrated bone marrow replacement by human AML cells in vehicle or THU + dT treated mice (>90% hCD45+), improved by decitabine alone (~85% hCD45+) but most by THU + decitabine (~35% hCD45+) and THU + dT + decitabine (~42% hCD45+) (Fig. 4b, c) (that is, dT did not add to the benefit from THU). Murine hematopoiesis was completely suppressed with vehicle or THU + dT (0% murine Cd45+), almost completely suppressed with decitabine-alone (~5% mCd45+) but preserved with THU + decitabine (~40% Cd45+) and THU + dT + decitabine (~27% Cd45+) (Fig. 4c). Hemoglobin and platelets were most suppressed, and white cells (peripheral leukemia) most elevated, with vehicle or THU + dT treatment, but only mildly to moderately suppressed with any of the decitabine containing regimens (Fig. 4d). Spleen weights and histology confirmed replacement by AML cells (with necrotic areas) with vehicle or THU + dT (Fig. 4e, f), less-so with decitabine-alone, and normal-appearance and lowest spleen weights with THU + decitabine and THU + dT + decitabine (Fig. 4e, f). We also evaluated the use of hydroxyurea to inhibit ribonucleotide reductase: hydroxyurea was administered on Day 1 before THU + decitabine on days 2 and 3: hydroxyurea, like dT, did not add further benefit to THU + decitabine (Supplementary Fig. S6A, B).

(c) *Frequent, distributed versus pulse-cycled schedules of administration*: DNMT1-depletion by decitabine or 5-azacytidine is S-phase dependent, suggesting frequent/distributed administration of THU + decitabine 2X/week, to increase chances of overlap between malignant S-phase entries and drug exposure windows, could be better than pulse-cycled THU + decitabine for 5 consecutive days every 4 weeks (Supplementary Fig. S6C) (such pulse-cycled schedules were created for anti-metabolite/cytotoxic therapy that requires several-week gaps to recover from the toxicity of treatment pulses). Vehicle-treated mice showed distress on Day 45, at which point all mice were euthanized or sacrificed for AML burden measurement. Vehicle-treated mice demonstrated median >95% bone marrow replacement by human CD45 + AML cells, decreased to median ~60% by THU + decitabine 5 days every 4 weeks, and decreased to median <10% by THU + decitabine 2X/week, with inversely corresponding murine Cd45+ cells (Supplementary Fig. S6D).

(d) *Exploit cross-priming by Dec and 5Aza for each other*: We compared head-to-head THU + decitabine 3X/week versus THU + 5-azacytidine 3X/week and found no difference in efficacy between these two treatments, using AML burden or time-to-distress end-points in separate experiments (Figs. 5, Supplementary Fig. S7). Then, since decitabine appears to cross-prime for 5-azacytidine activity by upregulating UCK2, while 5-azacytidine cross-primes for decitabine activity by upregulating DCK (Figs. 1–3, Supplementary Fig. S3), we alternated THU + decitabine with THU + 5-azacytidine week-to-week, and compared this to THU + decitabine or decitabine alone. Mice tail-vein innoculated with patient-derived AML cells (1 × 10^6^ cells/mouse) were randomized to (i) vehicle; (ii) decitabine alone; (iii) THU + decitabine; or (iv) THU + decitabine alternating with THU + 5-azacytidine week-to-week (Fig. 6a). Median survival (time-to-distress) was best with THU + decitabine/THU + 5-azacytidine (221 days) versus THU + decitabine (180 days), decitabine-alone (111 days) or vehicle (50 days) (Fig. 6b). Blood count stability during the weekly treatments was consistent with a noncytotoxic mechanism-of-action of the therapies (shown also previously [3, 26, 38–41]) (Fig. 6c). Eventual declines in hemoglobin and platelets were caused by progressive leukemia, shown by simultaneously increasing peripheral leukemia cells (increasing white cell counts) (Fig. 6c), and by flow cytometry analyses of bone marrows harvested after euthanasia (Fig. 6d). Alternating THU + decitabine with THU + 5-azacytidine in 4 week cycles, or simultaneous administration of THU + decitabine + 5-azacytidine, did not add benefit over THU + decitabine or THU + 5-azacytidine alone (Fig. 5, Supplementary Fig. S7). Thus, the benefit of alternating THU + decitabine with THU + 5-azacytidine depended on timing of alternation.

### Mechanisms-of-resistance in mice

Bone marrow cells harvested at day 63, when leukemia-innoculated mice were doing well on-therapy, demonstrated that the treatments depleted DNMT1, with the greatest depletions with THU + decitabine alternating with THU + 5-azacytidine week-to-week (~65% DNMT1-depletion) versus THU + decitabine (~50%), decitabine alone (~35%) or vehicle (~15%) (Fig. 6e). By contrast, bone marrow AML cells harvested at the time of progressive leukemia on these therapies demonstrated failure to deplete DNMT1 as measured by flow-cytometry (Fig. 6e). These bone marrows also demonstrated significant upregulations of CDA and CAD, with the greatest upregulations in AML cells from mice that received alternating THU + decitabine/THU + 5-azacytidine (Fig. 6f).

## Discussion

Decitabine and 5-azacytidine are processed by pyrimidine metabolism into the DNMT1-depleting nucleotide Aza-dCTP (“Supplementary Discussion”), and here we found that expression changes in pyrimidine metabolism enzymes was how malignant cells avoided DNMT1-depletion to resist decitabine or 5-azacytidine in vitro, in mice and in patients. The enzyme expression changes begun as adaptive responses to nucleotide imbalances caused by off-target depletion of TYMS by decitabine and RRM1 by 5-azacytidine. TYMS, like DNMT1, methylates carbon #5 of the pyrimidine ring, the carbon that is substituted with a chemically active nitrogen in decitabine/5-azacytidine, although the substrate is dUMP for TYMS and DNA-incorporated dCTP for DNMT1. Decitabine is metabolized into analogs of both these substrates and thus depletes both TYMS and DNMT1. TYMS-depletion decreases dTTP that in turn increases dCTP, because dTTP allosterically inhibits reduction of CDP into dCDP by ribonucleotide reductase [34–36]. TYMS-inhibition by decitabine, and hence dTTP suppression/dCTP upregulation, has also been documented by others [34–36]. A portion of administered 5-azacytidine can also be processed into Aza-dUMP via a circuitous six catalytic steps, but a more direct off-target action, requiring only two catalytic steps, is to form an analog, Aza-CDP, of the ribonucleotide reductase substrate CDP, which then depletes the ribonucleotide reductase sub-unit RRM1 - off-target inhibition of ribonucleotide reductase by 5-azacytidine, and hence dCTP suppression, has also been reported by others [42]. In brief, differential effects of decitabine and 5-azacytidine on TYMS versus RRM1 drive dCTP levels in opposite directions, triggering distinct responses from pyrimidine metabolism: DCK is particularly important for preserving dCTP, shown by less dCTP in *DCK*-knockout cells (shown also by others [43]); hence, DCK is appropriately upregulated upon dCTP suppression by 5-azacytidine, observed also by others [15]. UCK2 on the other hand appears particularly important for dTTP maintenance, shown by less dTTP in *UCK2*-knockout cells. Therefore, UCK2 is upregulated by decitabine. CDA also contributes to dTTP maintenance, thus decitabine and other drugs that inhibit TYMS/lower dTTP also acutely upregulate CDA (Supplementary references).

This mode of resistance, that emerges organically from metabolic networks purposed for homeostasis, does not require genetic mutations, and consistent with this, several studies that have looked for correlations between MDS/AML mutations and decitabine/5-azacytidine resistance have generated inconclusive or contradictory results [4, 44–49]. Baseline expression levels of these enzymes may also not necessarily be predictive [50, 51] since the metabolic reconfigurations are molded by treatment. We found that the predictable trajectory of acute metabolic responses to the pro-drugs, however, enables outmaneuvering and even exploitation: (i) first, in PDX models of chemorefractory AML, scheduling decitabine administrations to avoid reactive troughs in DCK expression was notably superior to schedules that coincided with DCK troughs. (ii) Second, alternating decitabine with 5-azacytidine week-to-week, timed approximately to exploit priming of each pro-drug for the other (UCK2 and DCK are maximally upregulated ~96 h after decitabine and 5-azacytidine respectively), was significantly superior to administration of either pro-drug alone. The timing of alternation was critical—alternating the pro-drugs in 4 week cycles, or their simultaneous administration, did not add benefit over the single agents. (iii) Third, frequent-distributed administration schedules, to increase possibilities of overlap between malignant cell S-phase entries and drug exposure windows, was superior to pulse-cycled schedules; pulse-cycled schedules concentrate treatment in pulses of a few consecutive days separated by several-week gaps needed for recovery from cytotoxicity—such long gaps are not needed if decitabine or 5-azacytidine doses are selected for noncytotoxic DNMT1-depletion, as shown also in previous clinical trials [3, 4, 52]. Observations from others also support rationalization of treatment schedules to increase S-phase dependent DNMT1-depletion: RNA-sequencing analysis of patients’ baseline bone marrows found that a gene expression signature of low cell cycle fraction predicted non-response to pulse-cycled 5-azacytidine therapy [50], and regulatory approval of decitabine and 5-azacytidine to treat myeloid malignancies occurred after doses were lowered from initially evaluated, toxic high doses, then administered more frequently [1]. (iv) Fourth, adding THU, to inhibit the catabolic enzyme CDA that severely limits decitabine and 5-azacytidine tissue-distribution and half-lives, and that is rapidly upregulated by decitabine (and to a lesser extent 5-azacytidine) in vitro and in vivo, also extended decitabine or 5-azacytidine anti-AML efficacy in vivo. An important detail in such combinations was that the decitabine and 5-azacytidine doses were lowered to preserve a noncytotoxic DNMT1-targeting mode of action [3, 26, 40, 41]. Stated another way, dose-escalations of decitabine or 5-azacytidine are not a solution for resistance since this compromises therapeutic-index: AML cells indefinitely self-replicate/proliferate and therefore have the opportunity to be educated for resistance from repeated treatment exposures, but normal myelopoiesis proliferates and terminally differentiates in successive waves—each wave is treatment naïve and vulnerable to off-target anti-metabolite effects of high doses. The clinical tools to translate these preclinical observations are available, since combination formulations of decitabine with the CDA inhibitors THU or cedazuridine (a THU analog) are in clinical trials [4, 40, 53]; these oral drugs can produce lower decitabine *C*_max_ but longer half-life than with approved intravenous regimens of decitabine, to deplete DNMT1 without cytotoxicity and facilitate frequent-distributed ingestion, as well as counter auto-upregulation of CDA [4, 40].

CAD, the enzyme that initiates de novo pyrimidine synthesis, was downregulated acutely by decitabine or 5-azacytidine, likely because of terminal-differentiation of most of the AML cells, but was upregulated at stable resistance to decitabine. This was the only discrepancy we found between acute versus chronic metabolic reconfiguration. We did not find benefit in vivo from combining decitabine with dT or hydroxyurea to inhibit ribonucleotide reductase that is in the de novo pyrimidine, and purine, synthesis pathways. Others, however, have found promise in vitro adding specific inhibitors of de novo pyrimidine synthesis: pyrazofurin to inhbit uridine monophosphate synthetase [54], PALA to inhibit CAD [17] or leflunomide to inhibit dihydroorotate dehydrogenase [55], all augmented 5-azacytidine activity in vitro. As with CDA-inhibitors, reductions in doses of concurrently administered 5-azacytidine/decitabine will likely be required to preserve noncytotoxic DNMT1-targeting, since toxicities caused failure of previous clinical trials of pyrazofurin with high dose 5-azacytidine [56].

In sum, we found that resistance to decitabine and 5-azacytidine emerges from adaptive responses of the pyrimidine metabolism network. These network responses can be anticipated and exploited using simple, practical treatment modifications that preserve the vital therapeutic index of noncytotoxic DNMT1-depletion.

## Supplementary information

Supplementary material
