# Supplementary material for: Decitabine- and 5-azacytidine resistance emerges from adaptive responses of the pyrimidine metabolism network
Source: Leukemia. 2020 Aug 7;35(4):1023–36. doi: 10.1038/s41375-020-1003-x (PMC7867667; doi:10.1038/s41375-020-1003-x)
Supplement: Supplementary file 1 — Supplementary material [file 41375_2020_1003_MOESM1_ESM.docx]

**Decitabine- and 5-azacytidine-resistance emerges from adaptive responses of the pyrimidine metabolism network**

**SUPPLEMENT**

- **Supplementary Methods**
- **Supplementary References for Main Text Discussion**
- **Supplementary Discussion with References**
- **Supplementary Figures**

**Figure S1. Time to emergence of AML cells exponentially proliferating in presence of clinically relevant concentrations of decitabine or 5-azacytidine.**

**Figure S2. Decitabine-resistant AML cells preserved DNMT1 protein levels but 5-azacytidine was able to deplete-DNMT1 and cytoreduce these cells.**

**Figure S3. Time-course analyses of pyrimidine metabolism enzyme protein levels after single exposure to decitabine (Dec) 0.25 µM or 5-azacytidine 2.5 μM.**

**Figure S4. Impact of decitabine scheduling to avoid vs coincide with DCK troughs.**

**Figure S5. Impact of decitabine scheduling to avoid or coincide with DCK troughs.**

**Figure S6. Impact of (a) using hydroxyurea to inhibit ribonucleotide reductase, and (b) scheduling THU-decitabine for 5 days every 4 weeks instead of 2 days every week.**

**Figure S7. THU/decitabine *vs* THU/5-azacytidine *vs* THU/decitabine/5-azacytidine (given simultaneously).**

**SUPPLEMENTARY METHODS**

***Study approvals*.** Bone marrow samples for research were obtained from patients with myeloid malignancies on a study protocol approved by the Cleveland Clinic Institutional Review Board (Cleveland, Ohio), with written informed consent prior to inclusion in the study from all patients. Experiments using patient-derived xenotransplant models of AML were in accordance with a protocol approved by the Cleveland Clinic Institutional Animal Care and Use Committee (Cleveland, Ohio)**.**

***Sources of cells and animals*.** AML cell line OCI-AML3 was purchased from DSMZ (Braunschweig, Germany), and THP1, K562 and MOLM13 cell lines were purchased from ATCC (Manassas, Virginia). The AML cell lines were authenticated, and then re-authenticated after selection for resistance to decitabine or 5-azacytidine (Genetica cell line testing, Burlington, NC, or Applied Biosystems, Foster City, CA). Mycoplasma testing was also performed on resistant cell lines. *DCK* and *UCK2* knock-out leukemia (HAP1) cells were engineered via Horizon Discoveries (Cambridge, United Kingdom). Primary AML cells for inoculation into NSG mice were collected with written informed consent on Cleveland Clinic Institutional Review Board approved protocol 5024. NSG mice were purchased from Jackson Laboratories (Bar Harbor, Maine).

***DNMT1 Immuno-detection and quantitation*:** Immunohistochemistry (IHC) was performed on decalcified and formalin-fixed paraffin embedded bone marrow biopsy sections (4μm) and on positive and negative controls (parental and DNMT1-KO HCT116 cells). Antibodies used were mouse polyclonal anti-Dnmt1 (Abcam #ab19905, Cambridge, MA), 1:200 dilution for 32 minutes at room temperature, performed with Ventana Discovery using OmniMap detection and a high pH tris-based buffer (Cell Conditioning 1, Ventana #950-124). Nuclei positive for DNMT1 were identified and quantified in high resolution, large field-of-view images per ImageIQ algorithms (Image IQ Inc., Cleveland, OH) after segmentation of images and subtraction of bone as we have previously described[^4^](#_ENREF_4).

DNMT1-protein measurement by flow cytometry was performed as we have previously described [^40^](#_ENREF_40) using unlabeled anti-Dnmt1 antibody [EPR 3522] (0.0625 µg/test; Abcam; catalog no. ab92314) as the primary antibody

***DNA isolation, reverse transcription (RT) and real-time PCR.*** As we have previously described [^58^](#_ENREF_58). Primer sequences were:

| **Gene** | **Primer (Forward)** | **Primer (Reverse)** |
| --- | --- | --- |
| CAD | 5’-CTGACTTCTACACTGAGCATGG-3’ | 5’-CACGCATTGACAGGTTAATCAC-3’ |
| CDA | 5’-AAGGGTACAAGGATTTCAGGG-3’ | 5’-ACAATATACGTACCATCCGGC-3’ |
| DCK | 5’-AAGCTGCCCGTCTTTCTC-3’ | 5’-ACCACTTCCCAATCTTCACAC-3’ |
| UCK2 | 5’-ATCCCCGTGTATGACTTTGTC-3’ | 5’-CTTCATCTGGAACAGGTCTCG-3’ |
| GAPDH | 5’-ACATCGCTCAGACACCATG-3’ | 5’-TGTAGTTGAGGTCAATGAAGGG-3’ |

**1D SDS-polyacrylamide gel electrophoresis and Western blot analysis.** Were performed as we have previously described [^58^](#_ENREF_58). Antibodies used were:

| **Primary Antibodies Used** | | |
| --- | --- | --- |
| **Catalogue#** | **Name** | **Company** |
| ab13537 | DNMT1 antibody | Abcam |
| sc-393098 | dCK Antibody (H-5), Mouse | Santa Cruz |
| ab104731 | Anti-UCK2 antibody, Rabbit | ABCAM |
| 12662 | Phospho-CAD (Ser1859) | Cell Signaling |
| 11933 | CAD Antibody | Cell Signaling |
| GTX108663 | DCTD antibody | GeneTex |
| sc-390945 | TYMS Antibody (C-5) | Santa Cruz |
| sc-377415 | RRM1 Antibody (A-10) | Santa Cruz |
| sc-398294 | RRM2A Antibody (A-5) | Santa Cruz |
| PRS2383-100UG | Anti-P53R2 antibody (RRM2A) | Sigma-Aldrich |
| ab82347 | Anti-CDA antibody | ABCAM |
| sc-374015 AF488 | Lamin B1 Antibody (B-10) Alexa Fluor® 488 | SCBT |
| sc-47724 AF647 | GAPDH Antibody (0411) Alexa Fluor® 647 | SCBT |
| F3022-.2mL | Actin-FITC | Sigma-Aldrich |
| A304-543A | Rabbit anti-CTPS1 Antibody | Bethyl Lab |
| **Secondary Antibodies Used** | | |
| Catalogue# | **Name** | **Company** |
| A32735 | Goat anti-Rabbit IgG (H+L) Highly Cross-Adsorbed Secondary Antibody, Alexa Fluor Plus 800 | Invitrogen |
| A32730 | Goat anti-Mouse IgG (H+L) Highly Cross-Adsorbed Secondary Antibody, Alexa Fluor Plus 800 | Invitrogen |
| 12004158 | StarBright™ Blue 700 Goat Anti-Mouse IgG, 400 µl | Biorad |
| 12004161 | StarBright™ Blue 700 Goat Anti-Rabbit IgG, 400 µl | Biorad |

Fluorescent images were collected using Biorad’s ChemiDoc system and processed with Image lab.

***Giemsa staining of cells***. As we have previously described[^58^](#_ENREF_58).

***Flow Cytometry Analyses for human and murine CD45****.* As we have previously described[^58^](#_ENREF_58)^,^ [^59^](#_ENREF_59). Antibodies used were monoclonal anti-human CD45 (clone HI30, cat. No 304016, Biolegend, 1:100) and monoclonal anti-mouse CD45 (Clone 30-F11, cat. No 1031066, Biolegend, 1:100).

***Preparation and analysis of dNTP and NTP extracts*:** Cells were washed twice with ice-cold 1X PBS, and counted. To lyse cells, precipitate proteins and extract nucleotides and nucleosides, for each 5-10 million cells, 250 μL of 80% acetonitrile/water was added, and the cells were incubated on ice for 15min. After incubation, the suspension was centrifuged at 140K rpm for 5min. The supernatant from this first extraction was transferred to a clean tube. The remaining pellet was extracted again with fresh 80% acetonitrile/water, and supernatant from both extractions was combined, and then evaporated to dryness using a centrifugal evaporator. *LCMS/MS*: 1mM Internal standards (13C9 15N3MP and d 13C9 15N3TP) solution (25mM ammonia acetate, 10mM DMHA, pH 8.0) was used to suspend Nucleotides and nucleosides extract. HPLC separation was carried on an ACQUITY UPLC HSS T3 Column, 100Å, 1.8 µm, 2.1 mm X 150 mm. A stepwise gradient program was applied with mobile phase A (25mM Ammonia Bicarbonate, 10mM DMHA, pH 8.0) and mobile phase B (60% Acetonitrile/water). The HPLC was interfaced with Thermofisher Quantiva triple quadruple mass spectrometer. The mass spectrometer was operated in MRM mode with optimized MRM transitions for each analyte. *Data analysis*: Xcalibur was used to process and quantify raw data. Briefly, a processing method was built using MRM transitions and peak retention times from standards. All samples were processed with the same method to generate integrated total ion intensity (integrated peak area) for each analyte. Manual inspection was performed to confirm the peak assignment and integration. The final report value was normalized to the internal standards and total number of cells used to generate the extract.

***Treatment of a patient-derived xenotransplant model of treatment-resistant AML.*** Patient-derived primary AML cells from a patient with AML that had progressed on standard chemotherapy then decitabine salvage therapy, were transplanted by tail-vein injection (1.0 x10^6^/mouse) into non-irradiated 6-8 week old NSG mice. Mice were anesthetized with isofluorane before transplantation. Mice were randomized to different treatments on Day 9 - 25 after inoculation, balanced only by sex and no other criteria. Doses of drugs used were: intra-peritoneal tetrahydrouridine (THU) 10 mg/kg given intra-peritoneal up to 3X/week; subcutaneous decitabine 0.2 mg/kg up to 3X/week (or 0.1 mg/kg when combined with THU); subcutaneous 5-azacytidine 2 mg/kg up to 3X/week (or 1 mg/kg when combined with THU); intra-peritoneal dT 2 g/kg up to 2X/week. Tail-vein blood samples for blood count measurement by HemaVet were obtained prior to leukemia inoculation, and at intervals thereafter as indicated in the figures. Mice were observed daily, including by veterinary staff blinded to treatment allocation, for signs of pain or distress (detailed in the Animal Protocol), e.g., weight loss that exceeded 20% of initial total body weight, lethargy, vocalization, loss of motor function to any of their limbs, and were euthanized by an IACUC approved protocol if such signs were noted.

***Statistics***. Assuming a rate of lethal AML in vehicle-treated mice to be 100% versus 30% in drug-treated mice, 6 weeks after AML cell inoculation by tail-vein into non-irradiated NSG mice, comparing these proportions with an alpha of 0.05 and single-sided power of 0.8, the required sample size in each group with equal allocation was 7 (Fishers Exact method (<https://stattools.crab.org/>). If early data indicated the treatment effect sizes were larger than this initial estimate, sample size was reduced to 5 mice per treatment group for subsequent experiments, in accordance with RRR (refine, reduce, replace) principles.

Tumor burdens were compared using non-parametric tests, and survival curves by the Log-rank test. Wilcoxon rank sum, Mann Whitney, and t tests were 2-sided unless otherwise stated because experiments were confirming prior literature observations (dCTP level analyses), and performed at the 0.05 significance level or lower (Bonferroni corrections were applied for instances of multiple parallel testing). Standard deviations (SD) and inter-quartile ranges (IQR) for each set of measurements were calculated and represented as y-axis error bars on each graph. Data-points/distributions from in vitro experiments are from biological replicates, and data-points from clinical and murine in vivo experiments are from individual patients/animals as indicated by sample sizes described in figure legends.

Graph Prism (GraphPad, San Diego, CA) or SAS statistical software (SAS Institute Inc., Cary, NC) was used to perform statistical analysis including correlation analyses.

**SUPPLEMENTARY REFERENCES for MAIN TEXT DISCUSSION**

CDA also contributes to dTTP maintenance^1-4^, thus CDA upregulation is also an appropriate compensatory response to dTTP suppression by decitabine, seen not just with decitabine, but also with other drugs that inhibit TYMS^5-10^.

1. de Saint Vincent BR, Dechamps M, Buttin G. The modulation of the thymidine triphosphate pool of Chinese hamster cells by dCMP deaminase and UDP reductase. Thymidine auxotrophy induced by CTP in dCMP deaminase-deficient lines. J Biol Chem 1980 Jan 10; 255(1): 162-167.

2. Moro-Bulnes A, Castillo-Acosta VM, Valente M, Carrero-Lerida J, Perez-Moreno G, Ruiz-Perez LM, et al. Contribution of Cytidine Deaminase to Thymidylate Biosynthesis in Trypanosoma brucei: Intracellular Localization and Properties of the Enzyme. mSphere 2019 Aug 7; 4(4).

3. Neuhard J. Pyrimidine nucleotide metabolism and pathways of thymidine triphosphate biosynthesis in Salmonella typhimurium. Journal of bacteriology 1968 Nov; 96(5): 1519-1527.

4. Andersen L, Kilstrup M, Neuhard J. Pyrimidine, purine and nitrogen control of cytosine deaminase synthesis in Escherichia coli K 12. Involvement of the glnLG and purR genes in the regulation of codA expression. Archives of microbiology 1989; 152(2): 115-118.

5. Momparler RL, Laliberte J. Induction of cytidine deaminase in HL-60 myeloid leukemic cells by 5-aza-2'-deoxycytidine. LeukRes 1990; 14(9): 751-754.

6. Mameri H, Bieche I, Meseure D, Marangoni E, Buhagiar-Labarchede G, Nicolas A, et al. Cytidine Deaminase Deficiency Reveals New Therapeutic Opportunities against Cancer. Clin Cancer Res 2017 Apr 15; 23(8): 2116-2126.

7. Meyers R, Malathi VG, Cox RP, Silber R. Studies on nucleoside deaminase. Increase in activity in HeLa cell cultures caused by cytosine arabinoside. J Biol Chem 1973 Sep 10; 248(17): 5909-5913.

8. Chen P, Aimiuwu J, Xie Z, Wei X, Liu S, Klisovic R, et al. Biochemical modulation of aracytidine (Ara-C) effects by GTI-2040, a ribonucleotide reductase inhibitor, in K562 human leukemia cells. The AAPS journal 2011 Mar; 13(1): 131-140.

9. Steuart CD, Burke PJ. Cytidine deaminase and the development of resistance to arabinosyl cytosine. NatNew Biol 1971; 233(38): 109-110.

10. Roberts D, Loehr EV. Depression of thymidylate synthetase activity in response to cytosine arabinoside. Cancer Res 1972 Jun; 32(6): 1160-1169.

**SUPPLEMENTARY DISCUSSION WITH REFERENCES**

We focused on DNMT1 as the molecular target of therapy for both pharmacology and cancer biology reasons. DNMT1-depletion by decitabine or 5-azacytidine has been demonstrated and studied by several groups. Briefly summarizing this data, DNMT1 is the maintenance methyltransferase that is naturally recruited to the DNA replication fork during S-phase. At the replication fork, DNMT1 directly interacts with the nucleotide form of decitabine and 5-azacytidine, Aza-dCTP, incorporated into the newly synthesized DNA strand. DNMT1, attempting to methylate the position #5 carbon of the pyrimidine ring, replaced with nitrogen in Aza-dCTP, is covalently bound and subsequently degraded^1, 2^. DNMT3A and DNMT3B are not naturally recruited to the replication fork. Therefore, DNMT3A/3B are minimally depleted, if at all, by decitabine treatment of cells^3-5^, even though by structural analyses, DNMT3A/3B like DNMT1, are expected to be able to interact with Aza-dCTP in DNA. In so far as DNMT3A/3B are depleted after decitabine or 5-azacytidine treatment of cells, it is possible that such downregulation could be a secondary to the terminal-differentiation/loss-of-proliferation that results from DNMT1-depletion in leukemia or cancer cell lines.

There are also cancer biology reasons for a therapeutic focus on DNMT1: in addition to its function as the maintenance methyltransferase, DNMT1 is recruited into lineage master transcription factor hubs as a corepressor (corepressors are proteins or protein complexes recruited by DNA binding factors to repress gene transcription)^6-10^. Leukemogenic genetic alterations, e.g., *RUNX1* mutations/translocations, *NPM1* mutations, skew composition of these lineage master transcription factor hubs towards DNMT1 and other corepressors that repress lineage-differentiation genes, and away from the coactivators (e.g., SWI/SNF factors) that normally would activate these genes^6, 7, 10-14^ (reviewed in^15^). In this way, forward-differentiation is disconnected from proliferation in lineage-progenitors, to create malignant self-replication (proliferation without differentiation) in a lineage-progenitor context in which self-replication normally does not occur^15^. This is our biological rationale for targeting DNMT1: inhibiting these corepressors aberrantly enriched in lineage master transcription factor hubs reconnects forward-differentiation to terminate malignant self-replication^15^. By contrast, we have neither found DNMT3A nor 3B in our comprehensive proteomic analyses of lineage master transcription factor hubs in leukemia or cancer cells^6, 9, 10^, and thus do not have a similar biology/biochemistry rationale to target DNMT3A or DNMT3B^15^.

Thus, we focused on DNMT1 as both a pharmacologically and biologically validated target of decitabine and 5-azacytidine therapy.

*REFERENCES FOR SUPPLEMENTARY DISCUSSION*

1. Schermelleh L, Haemmer A, Spada F, Rosing N, Meilinger D, Rothbauer U, et al. Dynamics of Dnmt1 interaction with the replication machinery and its role in postreplicative maintenance of DNA methylation. Nucleic Acids Res 2007; 35(13): 4301-4312.

2. Frauer C, Leonhardt H. A versatile non-radioactive assay for DNA methyltransferase activity and DNA binding. Nucleic Acids Res 2009 Feb; 37(3): e22.

3. Palii SS, Van Emburgh BO, Sankpal UT, Brown KD, Robertson KD. DNA methylation inhibitor 5-Aza-2'-deoxycytidine induces reversible genome-wide DNA damage that is distinctly influenced by DNA methyltransferases 1 and 3B. MolCell Biol 2008; 28(2): 752-771.

4. Patel K, Dickson J, Din S, Macleod K, Jodrell D, Ramsahoye B. Targeting of 5-aza-2'-deoxycytidine residues by chromatin-associated DNMT1 induces proteasomal degradation of the free enzyme. Nucleic Acids Res 2010; 38(13): 4313-4324.

5. Ghoshal K, Datta J, Majumder S, Bai S, Kutay H, Motiwala T, et al. 5-Aza-deoxycytidine induces selective degradation of DNA methyltransferase 1 by a proteasomal pathway that requires the KEN box, bromo-adjacent homology domain, and nuclear localization signal. MolCell Biol 2005; 25(11): 4727-4741.

6. Gu X, Hu Z, Ebrahem Q, Crabb JS, Mahfouz RZ, Radivoyevitch T, et al. Runx1 regulation of Pu.1 corepressor/coactivator exchange identifies specific molecular targets for leukemia differentiation therapy. J Biol Chem 2014 May 23; 289(21): 14881-14895.

7. Hu Z, Gu X, Baraoidan K, Ibanez V, Sharma A, Kadkol S, et al. RUNX1 regulates corepressor interactions of PU.1. Blood 2011 Jun 16; 117(24): 6498-6508.

8. Hu Z, Negrotto S, Gu X, Mahfouz R, Ng KP, Ebrahem Q, et al. Decitabine maintains hematopoietic precursor self-renewal by preventing repression of stem cell genes by a differentiation-inducing stimulus. Mol Cancer Ther 2010 Jun; 9(6): 1536-1543.

9. Enane FO, Shuen WH, Gu X, Quteba E, Przychodzen B, Makishima H, et al. GATA4 loss of function in liver cancer impedes precursor to hepatocyte transition. J Clin Invest 2017 Sep 1; 127(9): 3527-3542.

10. Gu X, Ebrahem Q, Mahfouz RZ, Hasipek M, Enane F, Radivoyevitch T, et al. Leukemogenic nucleophosmin mutation disrupts the transcription factor hub that regulates granulomonocytic fates. J Clin Invest 2018 Oct 1; 128(10): 4260-4279.

11. Wang J, Saunthararajah Y, Redner RL, Liu JM. Inhibitors of histone deacetylase relieve ETO-mediated repression and induce differentiation of AML1-ETO leukemia cells. Cancer Res 1999 Jun 15; 59(12): 2766-2769.

12. Negrotto S, Ng KP, Jankowska AM, Bodo J, Gopalan B, Guinta K, et al. CpG methylation patterns and decitabine treatment response in acute myeloid leukemia cells and normal hematopoietic precursors. Leukemia 2012 Feb; 26(2): 244-254.

13. Ng KP, Ebrahem Q, Negrotto S, Mahfouz RZ, Link KA, Hu Z, et al. p53 independent epigenetic-differentiation treatment in xenotransplant models of acute myeloid leukemia. Leukemia 2011 Nov; 25(11): 1739-1750.

14. Saunthararajah Y, Sekeres M, Advani A, Mahfouz R, Durkin L, Radivoyevitch T, et al. Evaluation of noncytotoxic DNMT1-depleting therapy in patients with myelodysplastic syndromes. J Clin Invest 2015 Mar 2; 125(3): 1043-1055.

15. Velcheti V, Schrump D, Saunthararajah Y. Ultimate Precision: Targeting Cancer but Not Normal Self-replication. American Society of Clinical Oncology educational book American Society of Clinical Oncology Annual Meeting 2018 May 23; (38): 950-963.

**
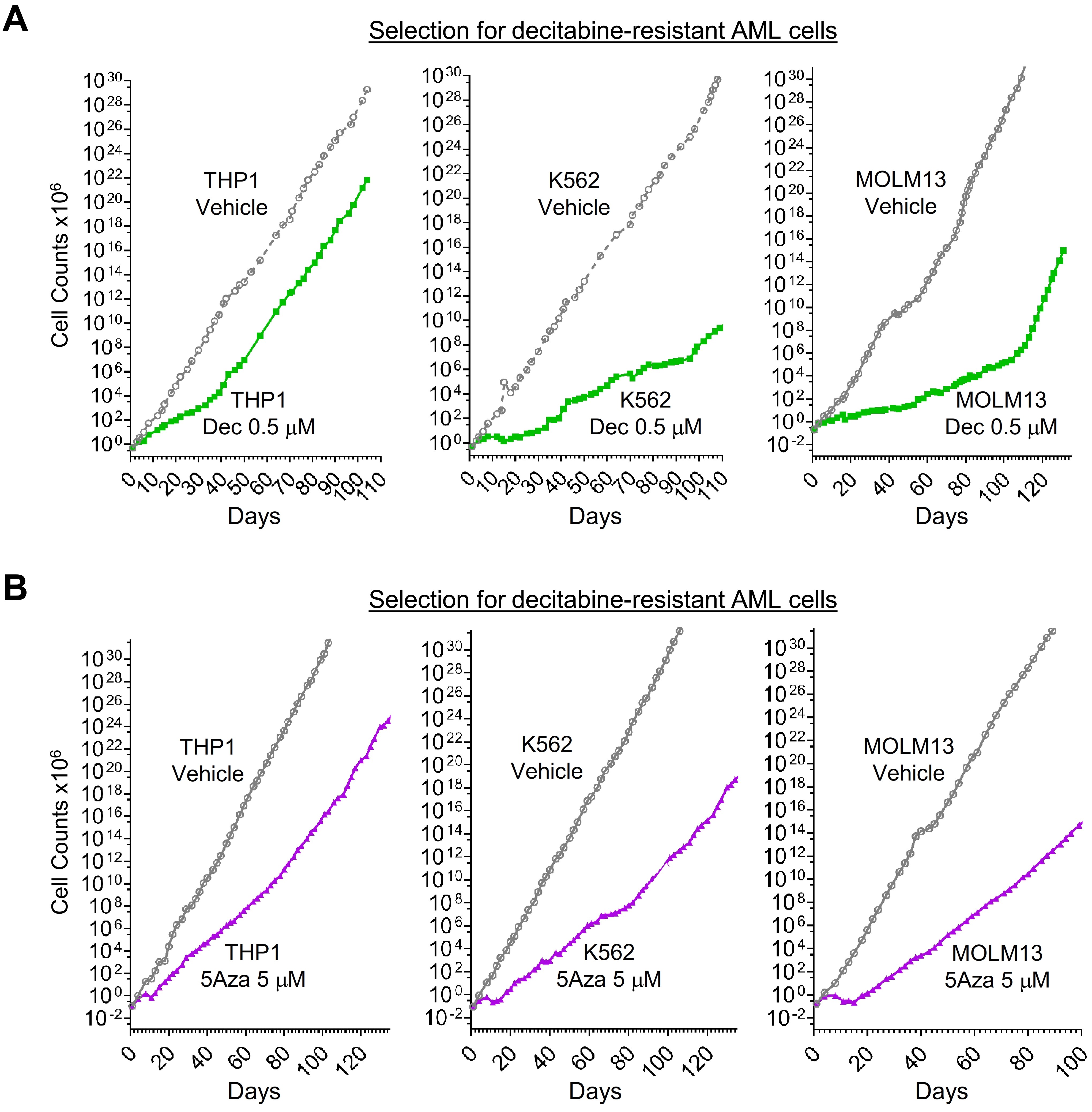
**

**Figure S1. A) Time to emergence of AML cells exponentially proliferating in presence of clinically relevant concentrations of decitabine (Dec).** Dec was added at a concentration of 0.25 μM with each replating until ~day 40 at which time-point the concentration was increased to 0.5 μM. Cell counts by automated counter. Resistant cell lines were re-authenticated using Applied Biosystems Cell Line Authentication services (Foster City, CA). **B)** **Time to emergence of AML cells exponentially proliferating in presence of clinically relevant concentrations of 5-azacytidine (5Aza).** 5Aza was added at a concentration of 2 μM with each replating until ~day 40 at which time-point the concentration was increased to 5 μM. Cell counts by automated counter. Resistant cell lines were re-authenticated using Applied Biosystems Cell Line Authentication services.

**
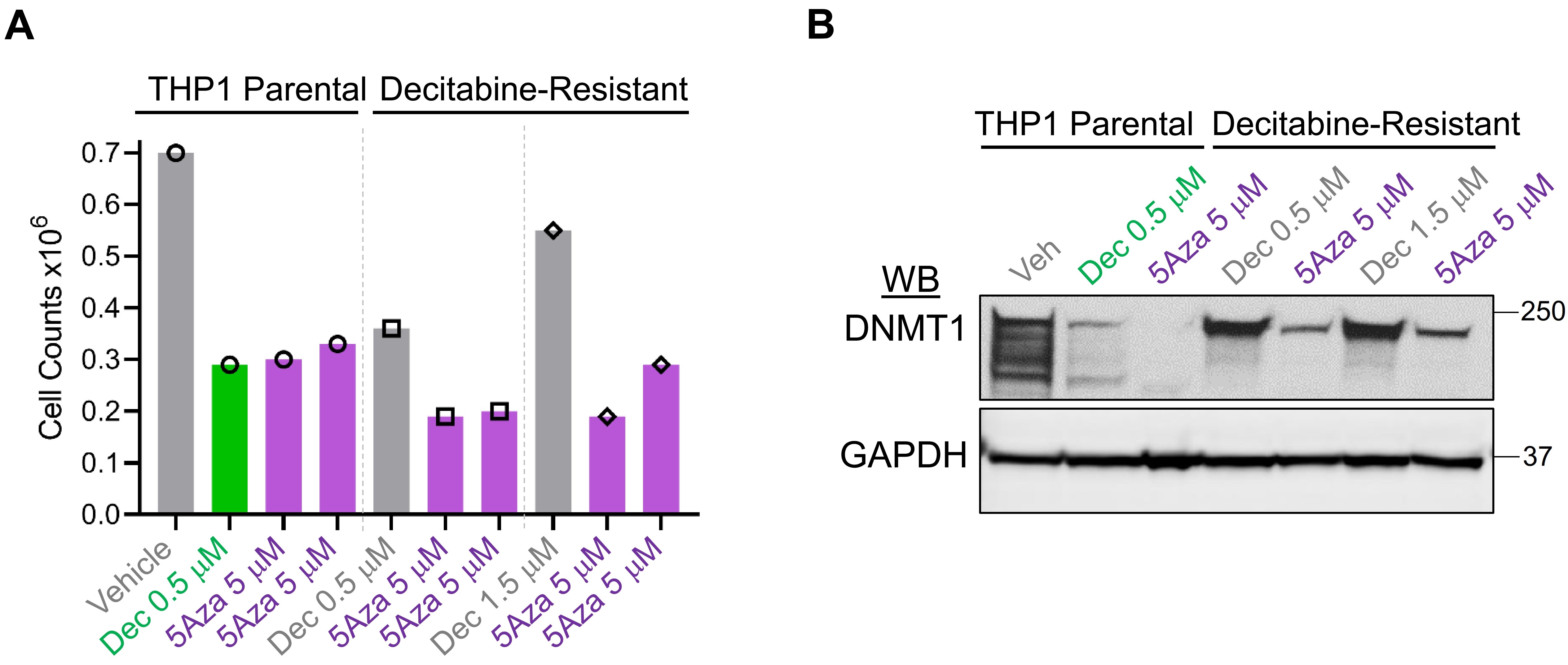
**

**Figure S2. Decitabine-resistant AML cells preserved DNMT1 protein levels but 5-azacytidine was able to deplete-DNMT1 and cytoreduce these cells. A) Cell counts of THP1 parental AML cells versus decitabine-resistant cells** (THP1 cells growing exponentially in the presence of decitabine 0.5 μM or 1.5 μM) 48 hours after addition of vehicle, decitabine or 5-azacytidine at the indicated concentrations. Decitabine-resistant THP1 AML cells were re-authenticated. **B) DNMT1 protein levels.** Measured 48 hours after addition of vehicle, decitabine or 5-azacytidine to the cells at the concentrations indicated. Western blot.


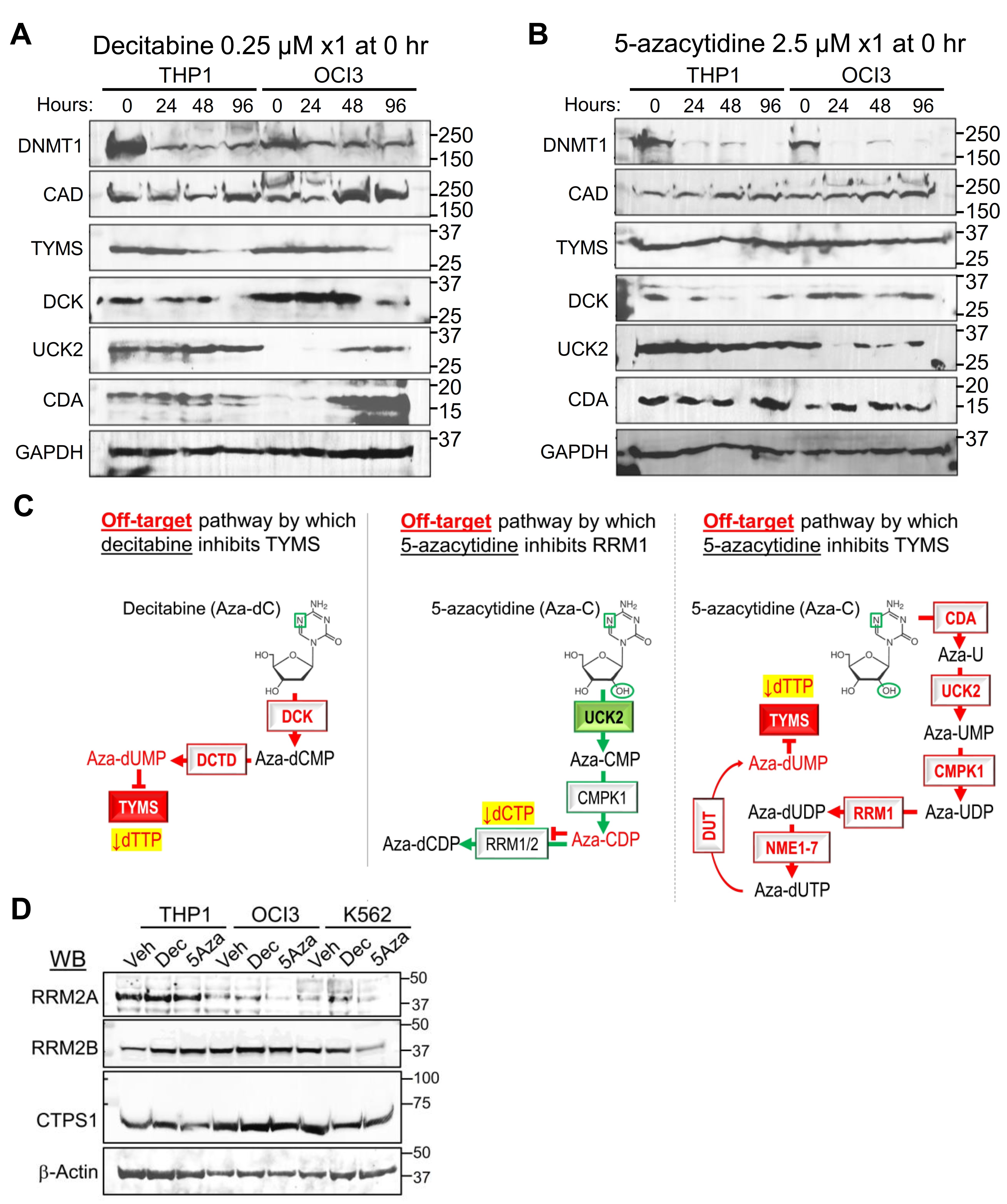


**Figure S3. A time-course analysis suggested that peak changes in pyrimidine metabolism enzyme protein levels occurred between 48-96 hours after a single exposure to decitabine (Dec) 0.25 µM or 5-azacytidine (5Aza) 2.5 μM. A) Western blots for DNMT1, UCK2, DCK, CDA, CAD, TYMS and GAPDH before and up to 96 hours after addition of decitabine (Dec) 0.25 µM** or **B) 5-azacytidine 2.5 μM to THP1 and OCI-AML3 cells** (added once at 0 hours). **C) Off-target pathways of Dec and 5Aza**. **D) Effects of Dec and 5Aza on RRM2A, RRM2B and CTPS1 protein levels.** Western blots 72 hours after addition of a single dose of Dec 0.25 µM or 5Aza 2.5 μM. Western blots were reproduced in biological replicates.


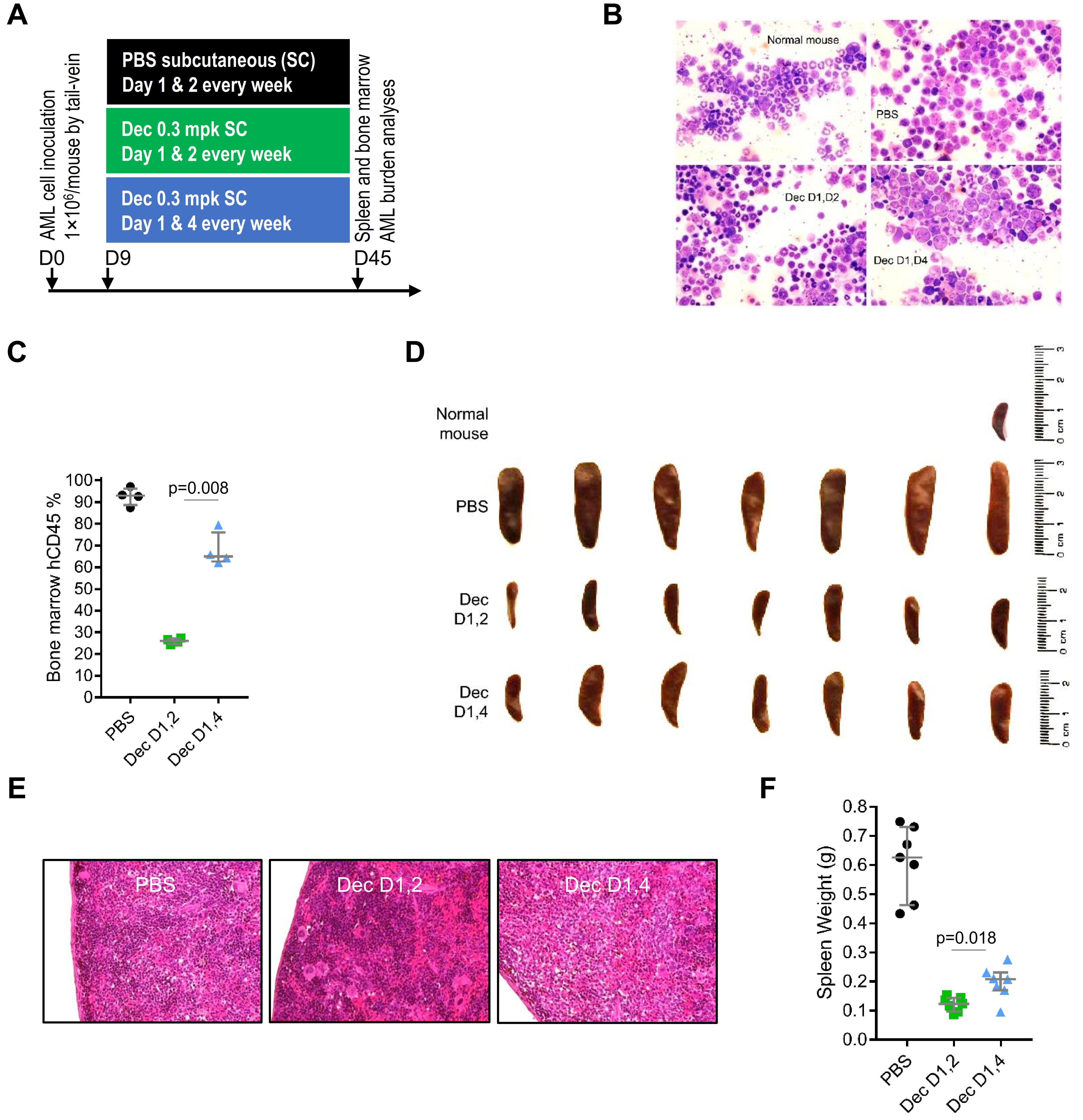


**Figure S4. Impact of decitabine scheduling to avoid vs coincide with DCK troughs (primary end-point: AML burden).** NSG mice were tail-vein inoculated with patient-derived AML cells (1×10^6^cells/mouse) and randomized on day 9 after inoculation to (i) PBS vehicle control; (ii) subcutaneous (SC) decitabine (Dec) 0.3 mg/kg (mpk) on Day 1 and 2 of each week (D1,2); (iii) Dec 0.3 mpk on Day 1 and 4 of each week (D1,4)(n=7/group). Mice were euthanized/sacrificed on day 45 when PBS treated mice showed signs of distress. **A) Experiment schema**; **B) Bone marrow cell cytospin and Giemsa-stain at Day 45**. Normal = normal NSG mouse bone marrow; Leica DMR microscope, 630X. **C) Percentage of human CD45+ (huCD45) positive cells in bone marrow.** Flow cytometry. Median ± IQR. p-value Mann-Whitney test 2-sided. 5 mice in each treatment group analyzed. **D)** **Spleens at Day 45**. Normal = spleen from normal NSG mouse. **E) Spleen histology**. Hematoxylin-Eosin stain of paraffin-embedded sections. Leica DMR microscope, 400X.**F) Spleen weights.** Median±IQR. p-value Mann-Whitney test 2-sided.


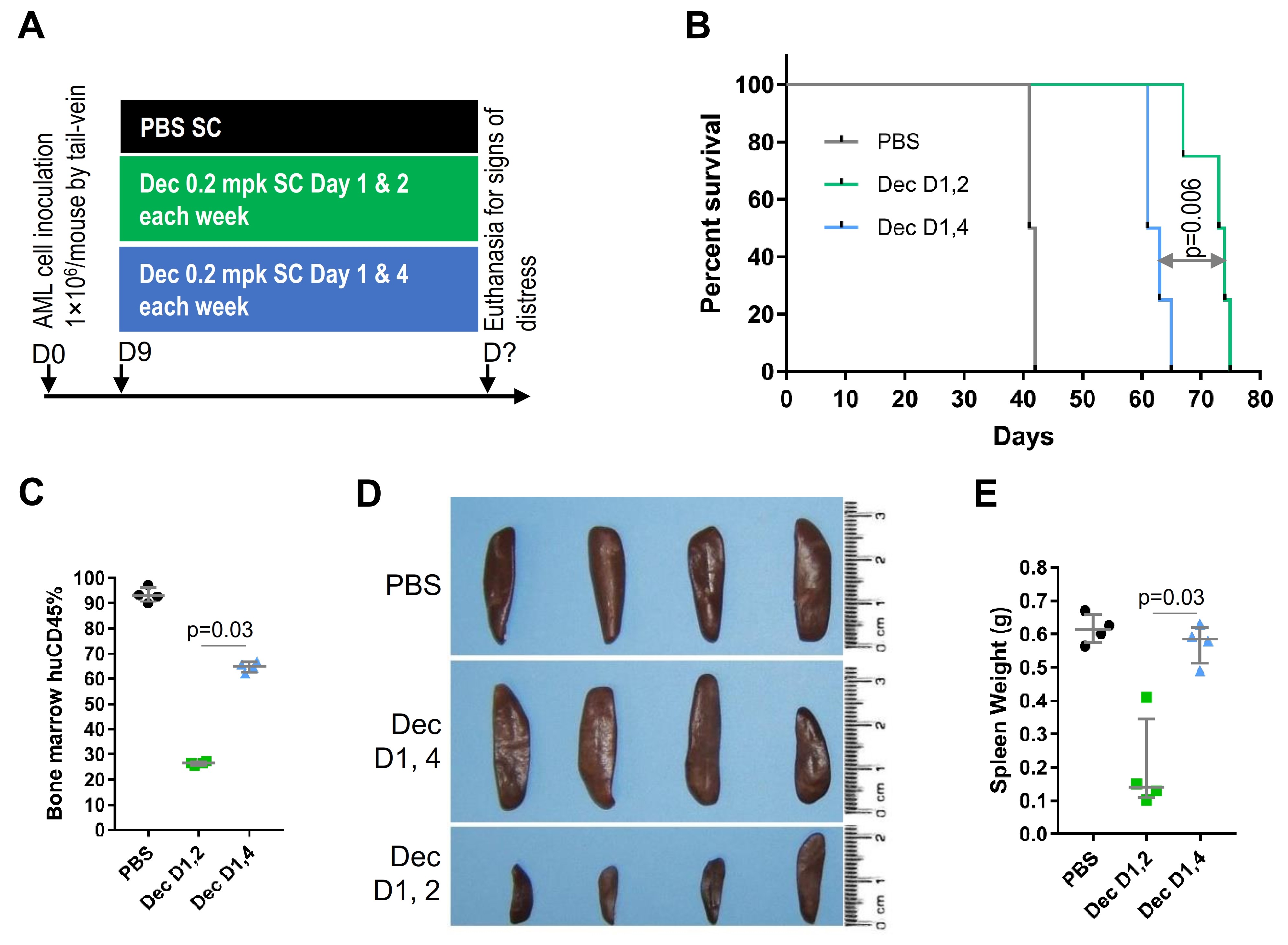


**Figure S5. Impact of decitabine scheduling to avoid or coincide with DCK troughs (primary end-point: time-to-distress/survival).** NSG mice were tail-vein inoculated with patient-derived AML cells (1×10^6^cells/mouse) and on day 9 after inoculation randomized to (i) PBS vehicle control; (ii) subcutaneous (SC) decitabine (Dec) 0.2 mg/kg (mpk) on Day 1 and 2 of each week (D1,2); (iii) Dec 0.2 mpk on Day 1 and 4 of each week (D1,4)(n=4/group). Mice were euthanized for signs of distress. **A) Experiment schema**; **B) Time-to-distress.** p-value Log-rank test. **C) Bone marrow human leukemia cell burden at time-of-distress**. Flow cytometry for human CD45+ cells. Median ± IQR. p-value Mann-Whitney test 2-sided. **D)** **Spleens at time-of-distress**. **E) Spleen weights at time-of-distress.** Median ± IQR. p-value Mann-Whitney test 2-sided.


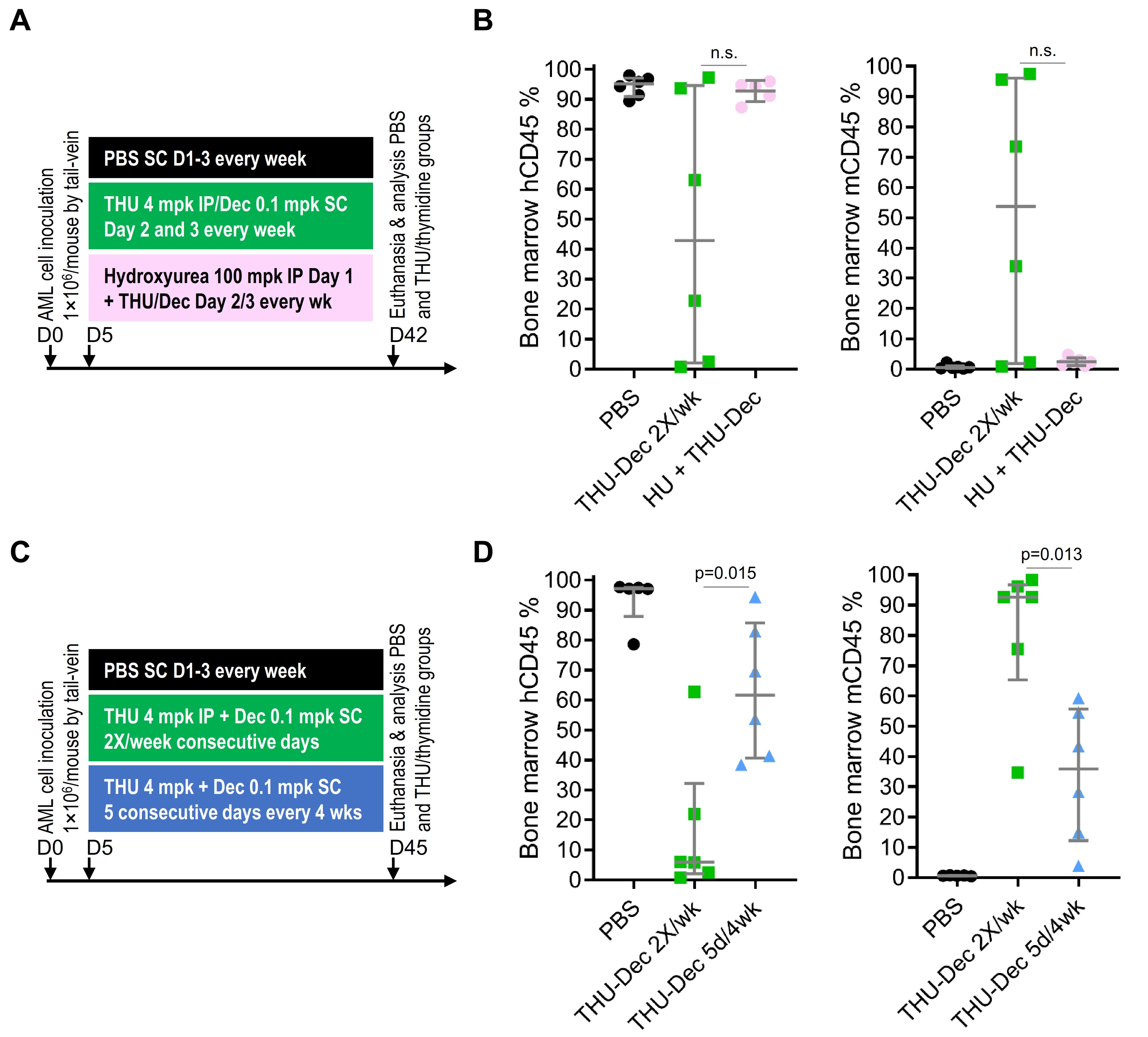


**Figure S6. The addition of hydroxyurea to inhibit ribonucleotide reductase did not augment THU-decitabine activity; Distributed administration of THU-decitabine 2X/week was superior to pulse-cycled administration of THU-decitabine for 5 consecutive days every 4 weeks.** NSG mice were tail-vein inoculated with patient-derived AML cells (1×10^6^cells/mouse) and on Day 5 after inoculation randomized to the treatments as shown (n=7/group). Mice were euthanized/sacrificed at ~Day 45 after innoculation. **A) Experiment schema to evaluate potential benefit of adding hydroxyurea to inhibit ribonucleotide reductase**; **B) Bone marrow human (hCD45) and murine (mCd45) myelopoiesis content.** Femoral bones flushed after termination of the experiment at the time PBS-treated mice developed signs of distress. Measured by flow-cytometry. Median±IQR. P-value 2-sided Mann-Whitney test. n.s. = not significant. **C) Experiment schema to compare metronomic administration of THU-decitabine 2X/week versus pulse-cycled administration of THU-decitabine for 5 consecutive days every 4 weeks**; **D) Bone marrow human (hCD45) and murine (mCd45) myelopoiesis content.** Femoral bones flushed after termination of the experiment at the time PBS-treated mice developed signs of distress. Measured by flow-cytometry. Median±IQR. P-value 2-sided Mann-Whitney test. n.s. = not significant.


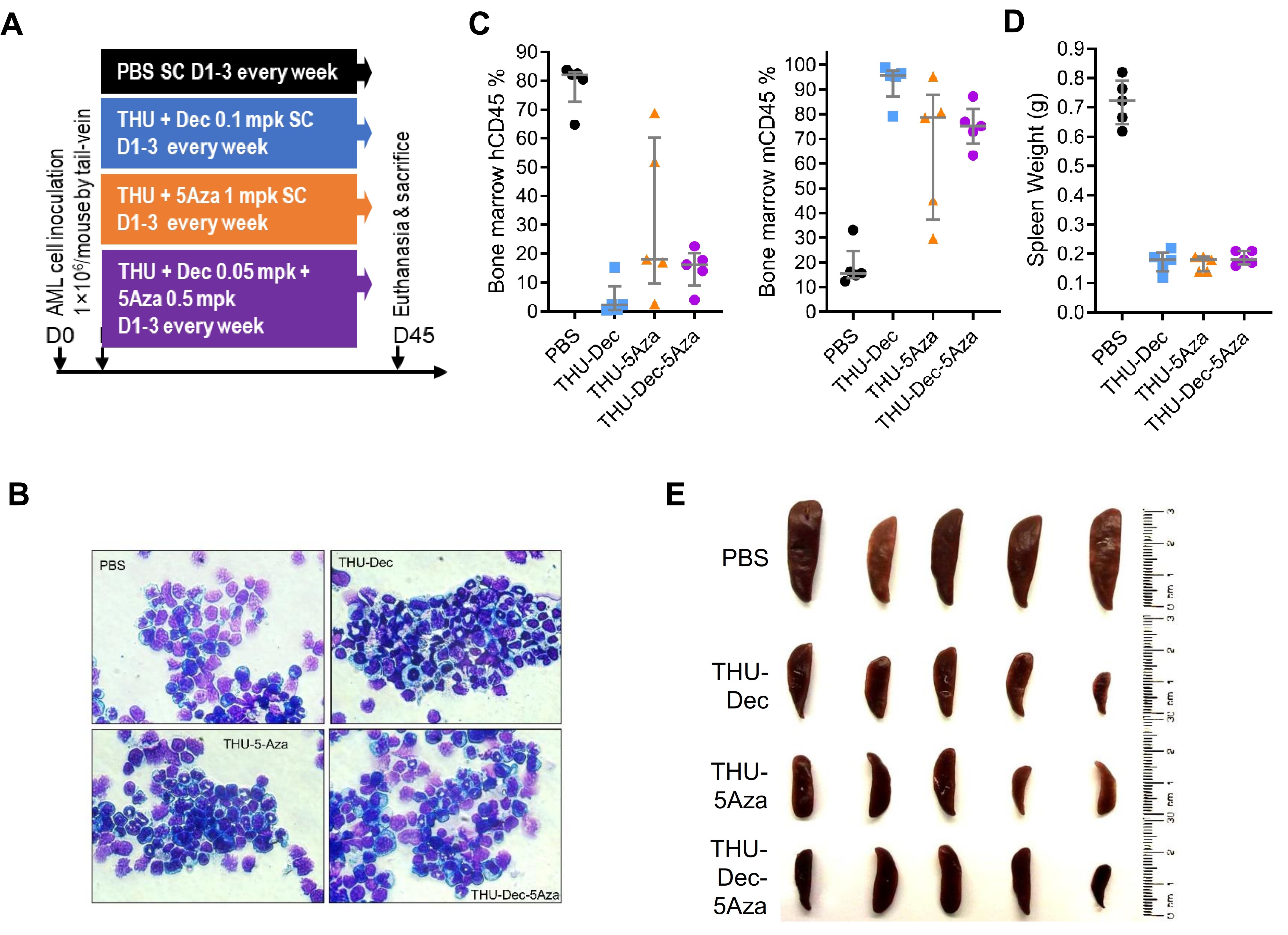


**Figure S7. THU/decitabine *vs* THU/5-azacytidine *vs* THU/decitabine/5-azacytidine.** NSG mice were tail-vein inoculated with patient-derived AML cells (1×10^6^cells/mouse) and on Day 9 after inoculation randomized to the treatments as shown (n=5/group). All mice were euthanized or sacrificed when the vehicle-treated group became distressed at Day 45. **A) Experiment schema**; **B) Giemsa stained cytospins of bone marrow cells.** Flushed from femoral bones after euthanasia. Magnification 630X. Leica DMR microscope.. **C) Bone marrow human (hCD45) and murine (mCd45) myelopoiesis content.** Measured by flow-cytometry. Median±IQR. **D)** **Spleen weights at time-of-distress/euthanasia**. Median±IQR. **E) Spleens**.
